# Variation of Cyclodextrin (CD) Complexation with Biogenic Amine Tyramine: Pseudopolymorphs of β-CD Inclusion vs. α-CD Exclusion, Deep Atomistic Insights †

**DOI:** 10.3390/ijms25147983

**Published:** 2024-07-22

**Authors:** Thammarat Aree

**Affiliations:** Department of Chemistry, Faculty of Science, Chulalongkorn University, Bangkok 10330, Thailand; athammar@chula.ac.th; Tel.: +66-2-2187584; Fax: +66-2-2187598

**Keywords:** cyclodextrin, tyramine, biogenic amines, catecholamine, pseudopolymorphs, X-ray analysis, DFT calculation, induced fit, hydrogen bonds, structural deformation

## Abstract

Tyramine (TRM) is a biogenic catecholamine neurotransmitter, which can trigger migraines and hypertension. TRM accumulated in foods is reduced and detected using additive cyclodextrins (CDs) while their association characteristics remain unclear. Here, single-crystal X-ray diffraction and density functional theory (DFT) calculation have been performed, demonstrating the elusive pseudopolymorphs in β-CD inclusion complexes with TRM base/HCl, β-CD·0.5TRM·7.6H_2_O (**1**) and β-CD·TRM HCl·4H_2_O (**2**) and the rare α-CD·0.5(TRM HCl)·10H_2_O (**3**) exclusion complex. Both **1** and **2** share the common inclusion mode with similar TRM structures in the round and elliptical β-CD cavities, belong to the monoclinic space group *P*2_1_, and have similar herringbone packing structures. Furthermore, **3** differs from **2**, as the smaller twofold symmetry-related, round α-CD prefers an exclusion complex with the twofold disordered TRM–H^+^ sites. In the orthorhombic *P*2_1_2_1_2 lattice, α-CDs are packed in a channel-type structure, where the column-like cavity is occupied by disordered water sites. DFT results indicate that β-CD remains elliptical to suitably accommodate TRM, yielding an energetically favorable inclusion complex, which is significantly contributed by the β-CD deformation, and the inclusion complex of α-CD with the TRM aminoethyl side chain is also energetically favorable compared to the exclusion mode. This study suggests the CD implications for food safety and drug/bioactive formulation and delivery.

## 1. Introduction

**Scheme 1:**
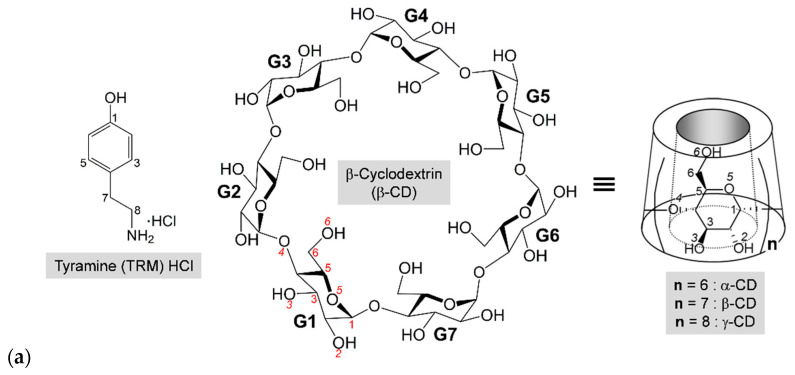

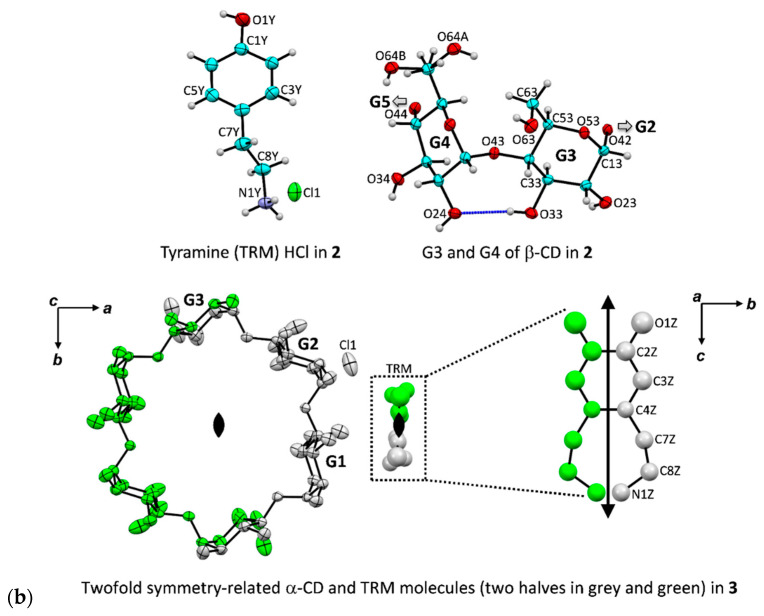
(**a**) Chemical structures and atom numbering schemes of tyramine (TRM) base/HCl and cyclodextrins (CDs). (**b**) X-ray-derived molecular structures of the inclusion complexes β-CD–TRM base (**1**), β-CD–TRM HCl (**2**), and the exclusion complex α-CD–TRM HCl (**3**); ORTEP plot at 30% probability level. In **2**, TRM base is protonated (TRM–H^+^) and counterbalanced by a fully occupied Cl^–^; its position is arbitrarily shown. Doubly disordered O64−H group of β-CD in **2** is indicated by letters A, B; both sites have a half occupancy factor. The connecting blue lines indicate the intramolecular, interglucose O2/O3∙∙∙O2/O3 H-bonds that stabilize the CD conformation. In **3**, one α-CD and 0.5 TRM–H^+^ are on the twofold-symmetry positions, respectively, at the O4 centroid and the bond midpoints of C2–C2(−*x* + 1, −*y* + 1, *z*) and C4–C4(−*x* + 1, −*y* + 1, *z*), along the *c*-axis (marked with 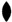, 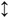). The doubly disordered TRM–H^+^ with equal occupancies of 0.25 is counterbalanced by 0.25 Cl^−^ in the intermolecular spaces.

Tyramine (TRM; 4-(2-aminoethyl)phenol, Scheme 1) is a biogenic monoamine derived from decarboxylation of the amino acid tyrosine. TRM is naturally prevalent in aged and fermented foods (cheese, chocolate, soy sauce, sauerkraut, kimchi), processed meats (sausages, hot dogs), and alcoholic beverages (wines, beers) [1]. TRM exhibits a two-sided coin effect on health: benefits and risks. It acts as a catecholamine releasing agent, maintaining the functionality of the brain and nervous system. However, high TRM intake can cause hypertension and migraines. A dose of 10 mg of TRM is associated with migraine onset. People experience migraine symptoms 1–12 h after ingesting TRM-rich diets [2,3]. The European Food Safety Authority (EFSA) recommended a safe level of tyramine intake of 600 mg per person per meal for healthy individuals and 50 mg for those receiving antidepressant (monoamine oxidase inhibitor (MAOI)) treatment [4], reducing the risk of dietary TRM–MAOI drug interactions—the “cheese effect” [5].

The molecular stability, water solubility, and bioavailability of food and drug molecules can be improved by cyclodextrin (CD) inclusion complexation; see a recent structural chemistry study on polyphenols in apples [6]. CDs, as macrocyclic polysaccharides, are versatile encapsulating agents with widespread applications in food, cosmetics, pharmacy, medicine, and the environment [7,8]. This is due to the unique amphipathic characteristics of the hollow truncated conical-shaped CD molecules. The two hydrophilic edges are lined by the secondary O2–H/O3–H groups on the wide side (head) and by the primary O6–H groups on the narrow side (tail). The hydrophobic portions are coated by various CH groups and ether-like O atoms, i.e., the central nanocavity by C3–H, C5–H and O4; and the outer surface by C1–H, C2–H, C4–H and O5 (Scheme 1). α-, β-, and γ-CDs comprising 6–8 glucose units typically feature bucket-like structures with cavity heights of 7.9 ± 1 Å and diameters of 4.7–5.3, 6.0–6.5, and 7.5–8.3 Å, which are stabilized by intramolecular, interglucose O2···O3 H-bonds with the separation distances of 2.902–3.150, 2.801–2.978, and 2.765–2.911 Å, respectively [9]. β-CD is the least soluble in water at room temperature (18.5 mg mL^−1^) because it is the most rigid and tends to form a dimer-based structure both in solid and solution [10,11]. Depending on the host–guest size compatibility and optimal intermolecular interactions, both total and partial inclusion complexes are crystallographically observed [12]. The β-CD cavity size is suitable for encapsulation of phenyl-containing guest molecules, as crystallographically evidenced from earlier studies on antidepressants [13] and various foods [6,14]. According to the EFSA based on the toxicological database [15], an acceptable daily intake of 5 mg/kg body weight per day of β-CD is suggested, while α- and γ-CDs are freely consumable [16].

CD inclusion complexation is conveniently characterized in aqueous solution by NMR, UV-visible, and fluorescence spectroscopy, albeit lacking atomistic details, particularly the intermolecular interactions underlying the complex stability. The literature on CD inclusion complexes with TRM is limited to the HCl form. Roy et al. [17,18] comprehensively investigated the inclusion complexes of TRM HCl with α- and β-CDs in aqueous solution and reported that both complexes have a unimolar host–guest ratio and are comparatively stable based on surface tension, conductivity, UV-visible, and NMR data. Furthermore, the NMR-predicted inclusion mode of both complexes is such that the TRM–H^+^ aromatic ring is buried in the CD cavity with the –NH_3_^+^ and OH groups are respectively situated near the wider and narrower rims [17,18]. The UV-visible-derived binding constants are 1067 M^−1^ (α-CD) and 1110 M^−1^ (β-CD) at 298 K, indicating that both complexes are comparatively moderately stable [17,18]. In contrast, the calorimetric titration-derived value of the 1:1 β-CD–TRM HCl is ~16 times smaller, 70 ± 2 M^−1^ at 298 K, which is significantly attributed to the O–H_TRM_···O_CD_ H-bond formation [19]. Additionally, the hydroxypropyl-β-CD–ferulic acid(FEA) inclusion complexes, as food additives, effectively decrease the TRM production in smoked horsemeat sausages, thus improving the food safety [20].

This work is inspired by the crystal structures of β-CD inclusion complexes with tricyclic antidepressant (TCA) amitriptyline in HCl salt form [13] and freebase form [21]. Although single crystals of both complexes are obtained from different laboratories using similar crystallization conditions (i.e., slow solvent evaporation of aqueous/aqueous–ethanol solution), their crystal symmetries, unit cell dimensions, and molecular and crystal structures are mostly the same; the root mean square deviation (rmsd) of structure superposition = 0.019 Å [13]. This triggers us to a curiosity: are these observations pertinent in other CD inclusion complexes with food, drug, and bioactive molecules? To definitively answer this question, we propose the following three hypotheses. (i) A guest molecule containing a phenyl ring and existing in freebase and HCl forms could be co-crystallized with β-CD, yielding good complex single crystals for X-ray analysis. As TRM is commercially available in both forms, it is selected as a case study. Furthermore, preliminary structural analysis revealed that β-CD in complex with TRM HCl deforms to an elliptical conformation, whereas β-CD in complex with TRM base retains a round conformation. (ii) Therefore, we envisage that the above unexpected observation of β-CD structural deviations from an annular conformation is due to the distinction of host–guest interactions between the two complexes. (iii) In contrast to the β-CD–TRM inclusion complex, the TRM aromatic ring is rather large to be entirely encapsulated within the α-CD cavity, making the formation of an α-CD–TRM inclusion complex energetically unfavorable and unlikely to occur. To assess postulates (i)–(iii), we attempted to crystallize the α- and β-CDs inclusion complex with TRM HCl/base as single crystals for X-ray analysis. To rationalize the structural distinction of the relevant complexes, the structure–energy relationship is established using density functional theory (DFT) calculation. To the best of our knowledge, this is the first comprehensive structural study on CD inclusion complexes with guest molecules in freebase and HCl salt forms, highlighting the rare phenomenon of pseudopolymorphism (i.e., the existence of distinct crystalline forms due to the different solvents/salts).

## 2. Results and Discussion

The atom labeling schemes of the inclusion complexes β-CD–TRM base (**1**), β-CD–TRM HCl (**2**), and α-CD–TRM HCl (**3**) are depicted in Scheme 1. Based on the carbohydrate nomenclature adopted, atoms C64–O64A/B represent the methylene C6–H_2_ bonded to the doubly disordered hydroxyl O6–H (sites A and B) of the glucose unit G4 of β-CD in **2**. TRM is labelled according to its IUPAC name, 4-(2-aminoethyl)phenol, and is distinguished by letters X, Y, and Z for the base and HCl forms in **1–3**. For a better understanding of the story, the discussion is outlined as follows. Crystallographic Section 2.1, Section 2.2 and Section 2.3 address how the induced-fit mechanism works in the β-CD–TRM base/HCl inclusion complexes and the α-CD–TRM HCl exclusion complex, the underlying intermolecular interactions stabilizing **1**–**3**, and the comparison of 3D arrangements and crystal contacts. Computational Section 2.4 explains the extent of host–guest structural changes and interactions contributing to the thermodynamic stability of various β-CD–TRM base inclusion complexes, and the theoretical plausibility of the α-CD–TRM base exclusion and inclusion complexes.

### 2.1. Structural Changes in β-CD Inclusion and α-CD Exclusion Complexes with TRM Base/HCl

**Figure 1:**
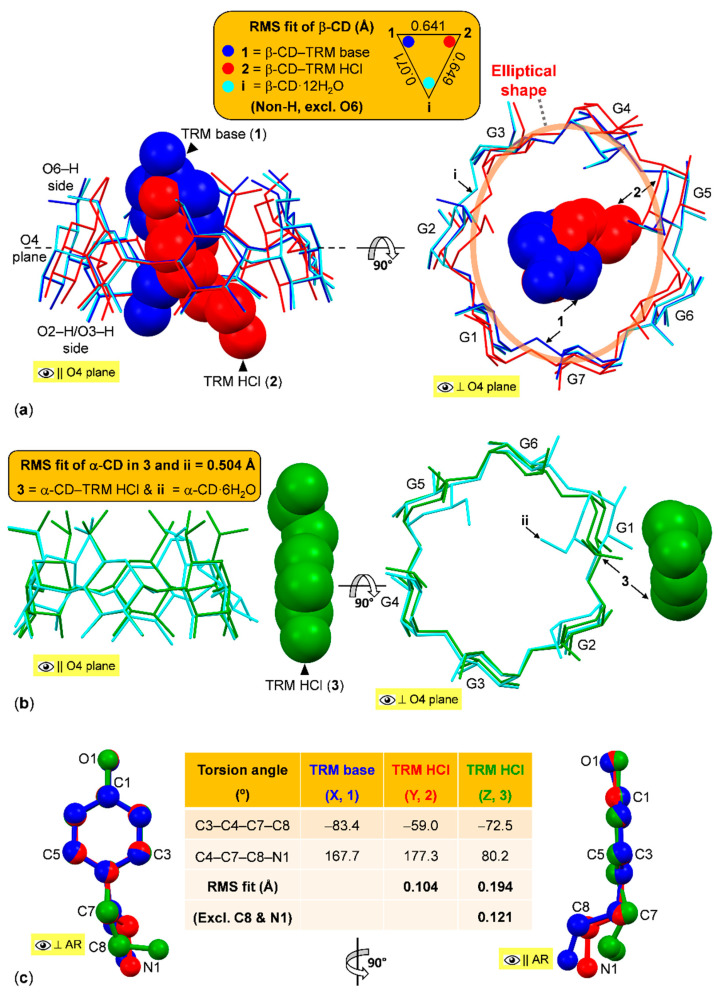
Structure overlays of (**a**) β-CD, (**b**) α-CD, and (**c**) TRM molecules in the inclusion complexes β-CD–TRM base (**1**), β-CD–TRM HCl (**2**), and the exclusion complex α-CD–TRM HCl (**3**), in comparison to the uncomplexed β-CD·12H_2_O (**i**; [23]) and α-CD·6H_2_O (**ii**; [24]), viewed with respect to the CD O4 plane and the TRM aromatic ring (AR). Only non-H atoms of the CD backbones (excluding O6) and the TRM rigid portion (excluding C8 and N1) are considered for the rms fit calculations (insets). Note the elliptical β-CD in **2** (light-orange line). Two torsion angles describing the TRM flexibility in **1**–**3** are listed (inset).

CDs comprise six to eight D-glucose residues linked via α-1,4-glycosidic bonds and inclined to molecular centroid, yielding an empty-bucket-like molecular structure. Usually, in the crystalline state, CD hydrates exist in an annular conformation stabilized by intramolecular, interglucose O3(*n*)···O2(*n* + 1) H-bonds (mainly), C6/O6(*n*)–H···O5(*n* + 1) H-bonds (partly and occasionally), and intermolecular O/N···O/N H-bonds with the surrounding CD, enclosed guest, and solvent molecules. To some extent, CDs adapt their structure for better inclusion complexation with various guests—a paradigm of the “induced-fit” mechanism [22]. Overall, β-CD accommodating TRM base (**1**) adopts a round conformation, mostly isostructural to the uncomplexed β-CD·12H_2_O (**i**; [23]), whereas β-CD encapsulating TRM HCl (**2**) is in a highly distorted round (viz., an elliptical round) shape. In contrast, a perfectly round macrocycle of the twofold symmetry-related α-CD is rather small to enclose the entire aromatic ring of TRM HCl in its cavity (**3**), thus forming an exclusion complex. It differs from the distorted round α-CD·6H_2_O (**ii**; [24]). This is globally evidenced by the structure overlays with different rms fits, 0.071 Å (**1** vs. **i**), 0.641 Å (**1** vs. **2**), 0.649 Å (**2** vs. **i**), and 0.504 Å (**3** vs. **ii**), Figure 1a,b. All non-H atoms of the CD skeletons are included in the rms fit calculations, disregarding the freely rotating O6 atoms.

To rationalize the apparent CD distinctions, we delve into the variations in the CD structural elements, although the composing glucose units are quite rigid (i.e., all are in similar ^4^*C*_1_ chair forms with normal spans of puckering parameters *Q*, 0.547–0.596 Å and *θ*, 0.0–10.6°) (Table S1a,b). The structural parameters describing the round conformations of β-CD {α-CD} involve the joining of seven {six} O4 atoms to form a regular heptagon {hexagon}, i.e., (i) the glucose tilt angles, (ii) the O4 fluctuation distances from their common mean plane, (iii) the ranges of O4(*n*)···O4(*n* − 1), [O4(*n*)···centroid] distances, and iv) the glycosidic torsion angles *φ* [O5(*n* + 1)–C1(*n* + 1)–O4(*n*)–C4(*n*)] and *ψ* [C1(*n* + 1)–O4(*n*)–C4(*n*)–C5(*n*)]. The corresponding key parameters of the distinct β-CDs (**1**, **i**, and **2**) and α-CDs {**3** and **ii**} are listed as follows: (i) (4.3–26.2°, 8.1–35.4°), {9.5–11.2°, 9.4–22.5°}; (ii) (–0.217 to 0.305 Å, –0.339 to 0.380 Å), {–0.016 to 0.018 Å, –0.135 to 0.057 Å}; and (iii) (0.242–0.264, [0.328–0.331] Å, 0.554, [1.028] Å); {0.091, [0.206] Å, 0.327, [0.708] Å} (Table S1a,b). The exception is the G1 tilt angle of **ii** that is largest (42.3°); its role on the α-CD structure is explained below.

**Figure 2:**
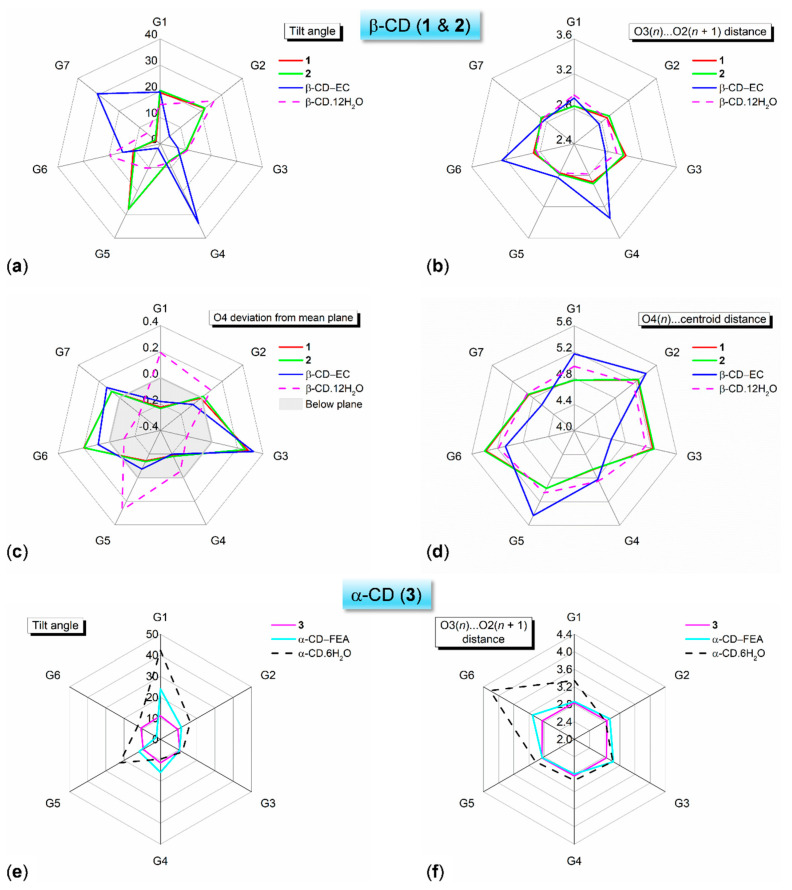
Radar plots showing the variations in (**a**,**e**) tilt angles, (**b**,**f**) O3(*n*)⋯O2(*n* + 1) distances, (**c**) O4(*n*) deviations, and (**d**) O4(*n*)⋯centroid distances in the glucose units G1–G6/G7 of α-/β-CD upon complexation with TRM base (**1**) and TRM HCl (**2**,**3**). For comparison, data from the inclusion complexes β-CD–(–)-epicatechin(EC) [14], α-CD–ferulic acid(FEA) [6], and the uncomplexed β-CD·12H_2_O [23], α-CD·6H_2_O [24] are included, and their glucose units are renumbered for the best fit; see also Table S1. In (**c**), the gray area indicates that the glucose units have O4 below their mean plane (negative values). Angles and distances are in ° and Å.

Note that glucose inclination not only relieves the ring strain, stabilizing the CD macrocycles, but also affects the interglucose O3(*n*)···O2(*n* + 1) H-bonds such that the tilt angles and the O3···O2 distances increase proportionally. In the inclusion complex β-CD–(–)-epicatechin(EC) [14], two diametrically opposed glucoses, G2 and G5, are largely inclined by 33.7° and 30.6°, disrupting the O32···O23 and O34···O25 H-bonds (O···O distances > 3.2 Å), resulting in a highly distorted β-CD. Similarly to **2**, the O36···O27 H-bond is broken (3.3-Å separation), and β-CD becomes elliptical due to the 35.4° tilting of G5, whereas for the round β-CDs in **1** and **i**, all 14 interglucose O3···O2 distances do not exceed 3.0 Å (Table S1). To better distinguish the structural parameters of CDs, radar plots of the key parameters are shown in Figure 2a–d. The spike (peak) positions of tilt angles, O3(*n*)···O2(*n* + 1) distances, O4(*n*) deviations, and O4(*n*)···centroid distances are similar. Larger values belong to the diametrically opposed glucoses; see those of the elliptical β-CD in **2**. Because glucose units G2, G5 (opposed positions) are largely inclined (Figure 2a), the O4(*n*) deviations, O4(*n*)···centroid, and O3(*n*)···O2(*n* + 1) distances of the adjacent glucoses G3/G4, G6/G7 (opposed positions), are most affected (Figure 2b–d). In addition, the strongly tilted glucose units G2, G5 (**1**, **2**) and G2, G6 (**i**) mostly point their O6–H groups inward to the CD cavity to H-bond with the surrounding TRM NH groups and water sites (Table 1, Tables S2 and S3), as indicated by torsion angles *χ* [C4–C5–C6–O6], ~±180° and *ω* [O5–C5–C6–O6], ~60°, Table S1. However, most O6–H groups direct outward from the CD cavity to H-bond with water molecules in the intermolecular spaces, the corresponding *χ*, ~60° and *ω*, ~–60°, Tables S1–S3.

In contrast to **2**, the structural changes of α-CD (**3**) become reverse upon complexation. The uncomplexed α-CD·6H_2_O (**ii**; [24]) adopts a distorted round conformation as the glucose units G1 and G5 are strongly tilted (42.3° and 22.5°) to the molecular centroid, facilitating the pointing inward of O61–H and O65–H groups to H-bond with a water molecule embedded in the cavity, Figure 1b and Table S1b. To properly interact with a guest molecule, the smaller α-CD ring adjusts its cavity for two possibilities of association: the mostly observed inclusion complexes, e.g., the α-CD–ferulic acid (FEA) [6]; and the rarely found exclusion complex (**3**). While in the former α-CD encapsulates the FEA side chain [6], in the latter α-CD outer surface interacts with the TRM aromatic ring. As a result, the broken O3(*n*)···O2(*n* + 1) H-bonds with O36···O21 and O31···O22 distances of 4.227 and 3.345 Å of the distorted round α-CD (**ii**) change to the normally round α-CD conformations in **3** and the FEA complex [6], as indicated by the O3(*n*)···O2(*n* + 1) distances < 3.2 Å and the glucose inclination angles < 20°. (Figure 2e,f and Table S1b).

### 2.2. How Are TRM Base/HCl Differently Stabilized While Associating with β-CD (Inclusion) and α-CD (Exclusion)?

**Figure 3:**
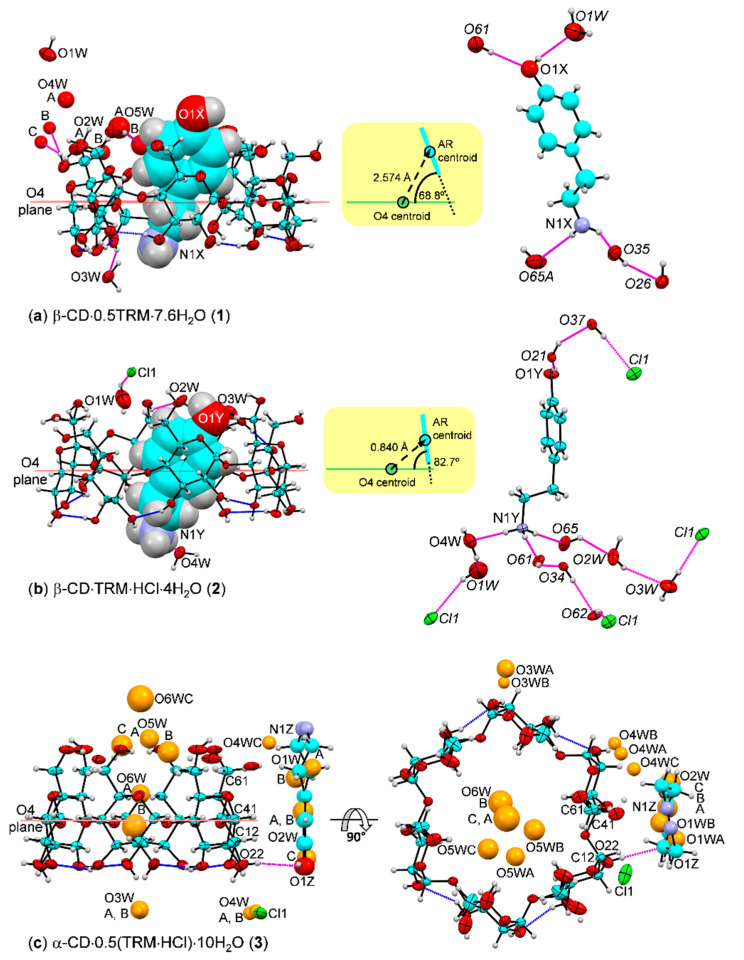
(Left) similar inclusion geometries of (**a**) a half-occupied TRM base in the round β-CD (**1**) and (**b**) a fully occupied TRM–H^+^ in the elliptical β-CD (**2**) in the crystalline state at 296 K. For clarity, TRM molecules are shown with a space-filling model and water sites with occupancies of 0.2–0.6 are disordered with 0.5 TRM inside and near the β-CD cavity are omitted (**1**). (**c**) The α-CD·0.5(TRM–H^+^Cl^−^) exclusion complex (**3**) in the solid state at 296 K; 10 water molecules distributed over 16 sites inside and outside the α-CD cavity are shown in orange balls. The intramolecular, interglucose O2/O3∙∙∙O2/O3 H-bonds within CD and intermolecular N/O–H∙∙∙O H-bonds are indicated by blue and magenta connecting lines, respectively. (Right) intermolecular N/O–H∙∙∙O/Cl H-bonds stabilizing TRM base (**1**) and TRM–H^+^ (**2**) in the distinct β-CD cavities (magenta lines). The OH groups and water molecules of the adjacent asymmetric units are labeled in italics. (Middle inset) inclusion geometries in **1** and **2** are shown with the β-CD O4-plane centroid relative to the TRM aromatic ring (AR) centroid and their interplanar angles. ORTEP diagrams are drawn at the 30% probability level.

TRM base (**1**) and TRM HCl (**2**, **3**) are similar in their rigid portion as indicated by a small span of rms fits of 0.104–0.194 Å; only non-H atoms, excluding C8 and N1, are considered for the calculation (Figure 1c). The relevant torsion angles C3–C4–C7–C8, C4–C7–C8–N1 are –83.4°, 167.7° (**1**), –59.0°, 177.3° (**2**) and –72.5°, 80.2° (**3**). The TRM molecular length (O1⋯N1 distance) of the HCl form (**2**) is 7.748 Å, which are 0.258 and 0.489 Å longer than those of the base form (**1**, 7.490 Å) and HCl form (**3**, 7.259 Å), respectively. The N1X (**1**), N1Y (**2**), and N1Z (**3**) atoms are away from the aromatic planes by 2.110, 1.487, and 0.078 Å, respectively; these are the deviations of N1X/Y/Z atoms from the least-squares aromatic plane (Figure 1c). Based on host–guest size complementarity, it is anticipated that (i) TRM can be totally enclosed in the larger β-CD cavity not the smaller α-CD cavity. (ii) The larger aminoethyl and smaller hydroxyl groups of TRM should be respectively placed near the wider O2–H/O3–H and narrower O6–H rims of the β-CD cavity, yielding an energetically preferable inclusion mode. The aminoethyl side chain is a more probable moiety to be embedded in the α-CD cavity, as observed for the α-CD–FEA [6]. Both assumptions are validated crystallographically in **1** and **2**, whose similar inclusion structures are stabilized by various intermolecular interactions (Figure 3a,b, left, right panels, and insets). Surprisingly, the α-CD–TRM HCl exclusion complex (**3**) is evident crystallographically (Figure 3c). These deepened atomistic details derived from single-crystal X-ray diffraction go beyond the spectroscopically deduced overall inclusion structures [17,18].

In **1**, the TRM base and water cluster (O5W–O13W) are half-occupied and mutually exclusive in the round β-CD cavity (Figure 3a). TRM base places its aromatic centroid 2.455 Å above and inclines 68.8° against the β-CD O4 plane and is kept in position by intermolecular O61–H⋯O1X–H⋯O1W and O65A⋯H2–N1X–H1⋯O35 H-bonds (Figure 3a and Table 1 and Table S2). In **2**, the well-ordered, protonated TRM–H^+^ is mostly vertically aligned (82.7°) in the elliptical β-CD cavity such that its aromatic centroid is 0.691 Å above the β-CD molecular plane and is stabilized by intermolecular O1Y–H⋯O21 and O4W⋯H2–N1Y–H1⋯O65(–H3⋯O61) H-bonds (Figure 3b and Table 1 and Table S2). Both ends of TRM–H^+^ (O1Y–H and N1Y–H_3_^+^) are further H-bonded with CD OH groups and water molecules until reaching the chlorides (Figure 3b). Similarly, note that in the crystals of the CD–drug inclusion complex hydrates, the salt anions (e.g., chloride, bromide) are not connected to the protonated amine groups [13], in contrast to the crystal of free TRM HCl, whose all H atoms of the NH_3_^+^ group are linked to Cl^−^ [25].

A question arises: to what extent the host–guest interactions in **1** and **2** are compared? In **2**, the mostly vertical pose and the position (inclusion depth) of TRM–H^+^ in the β-CD cavity induce a large tilting of two diametrically glucoses G2 and G5 (23.5° and 35.4°). Consequently, β-CD deforms to an elliptical shape, gripping the TRM–H^+^ aromatic ring through the host–guest C52–H⋯π and C56–H⋯π interactions (Figure 3b and Table 1 and Table S2). In contrast, a deeper insertion of the half-occupied TRM is the less favorable pose in the β-CD cavity (**1**). Therefore, β-CD remains round and just one intermolecular C51–H⋯π interaction is formed (Figure 3a and Table 1 and Table S1).

**Figure 4:**
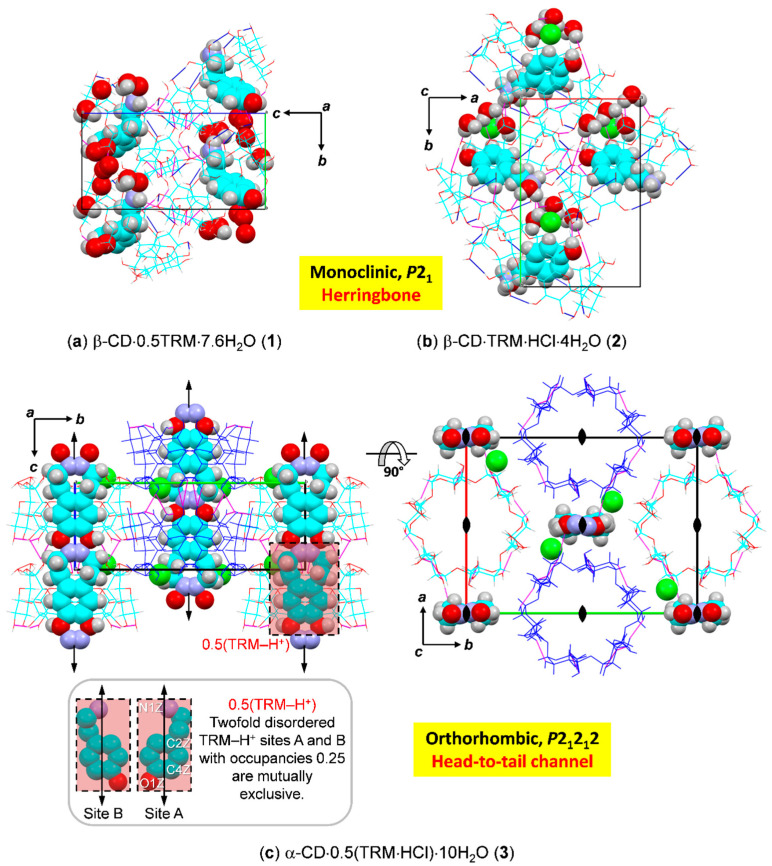
Crystal packing in a herringbone fashion of inclusion complexes (**a**) β-CD–TRM base (**1**) and (**b**) β-CD–TRM HCl (**2**), both in the monoclinic space group *P*2_1_. (**c**) Channel-type packing of the exclusion complex α-CD–TRM HCl (**3**), in the orthorhombic space group *P*2_1_2_1_2. CD macrocycles, TRM, water, and chloride are shown with the wireframe and space-filling models: C, cyan; O, red; N, purple; Cl, green; and H, light gray. In **1** and **3**, the disordered water sites are omitted for clarity. In **1** and **2**, the intramolecular, interglucose O2/O3∙∙∙O2/O3 H-bonds within CD and intermolecular N/O–H∙∙∙O/Cl H-bonds are indicated by blue and magenta connecting lines, respectively. In **3**, the intra- and intermolecular interactions are shown with magenta lines, the CD columns at *b* = 0.5 are marked in blue, and the twofold rotation symmetry along the *c*-axis is also labeled (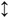, 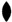). The twofold-symmetry, half-occupied TRM–H^+^ is explained in the inset. The intermolecular C–H···O interactions are not shown (Tables S2–S4).

The peculiar exclusion complex of α-CD·0.5(TRM–H^+^Cl^−^) (**3**) deserves further attention. The twofold symmetry-related α-CD cavity is somewhat small to accommodate the whole TRM molecule; thus, the round α-CD cavity anchors several disordered water sites instead (Figure 3c and Table 1 and Table S3). Surprisingly, the twofold disordered TRM–H^+^ sites A and B with equal occupancy factors of 0.25 are found in intermolecular spaces, outside the α-CD cavity. The TRM aromatic centroid is placed 0.070 Å above and 8.349 Å away from the α-CD molecular centroid (O4 plane center). TRM is perfectly vertically aligned (90.0°) against the CD molecular plane and its long molecular axis (passing through C7Z⋯O1Z) is slanted by 60° against the twofold rotation axis, giving rises to the twofold symmetry-related TRM sites A and B (Figure 3c and Figure 4c and Scheme 1. TRM is kept in position by intermolecular O22–H⋯O1Z H-bond and three C–H⋯π interactions (mainly) and crystal contacts of O_w_/O_CD_/C_TRM_–H⋯Cl interactions (partly), Figure 3c and Figure 4c and Table 1 and Table S3. This is an exclusion complex (**3**), which is rarely observed for CDs. Given examples of other exclusion complexes include dimethyl-β-CD–FEA [26] and β-CD–fenchene [27]. Thermodynamic stabilities of the inclusion and exclusion complexes of α-CD–TRM are rationalized and compared in Section 2.4.

### 2.3. 3-D Arrangements of Pseudopolymorphic β-CD–TRM Base/HCl Inclusion (***1**, **2***) and α-CD–TRM HCl Exclusion (***3***)

The isostructural molecular inclusion complex and comparable unit cell constants of **1** and **i** [23] suggest that the round β-CDs in both crystal lattices have the same 3-D arrangements—a herringbone pattern (Figure 4a), a typical packing structure of CD inclusion complexes with small molecules completely buried in the CD cavity [28]. Conversely, although the inclusion structures, unit cell volumes, and crystal symmetries of **1** and **2** are similar or identical, their unit cell lengths are somewhat different (see Section 3.2), suggesting that both complexes have similar 3-D arrangements. Gazing at the packing structures confirms that the elliptical β-CDs (**2**) are also packed in a similar herringbone pattern (Figure 4b). In **1** the β-CD molecular plane makes an angle of 42.3° against the twofold-screw axis along the *b*-axis, and in **2** the corresponding angle is larger, 60.0° (Figure 4a,b). Consequently, **2** has greater numbers of intermolecular O2/O3∙∙∙O6 H-bonds compared to **1** (Tables S2 and S3). In both cases, the intermolecular N/O–H∙∙∙O/Cl and C–H∙∙∙O interactions involve hydration water and the hydrochloride salt (Tables S2 and S3). The β-CD–TRM base/HCl inclusion complexes existing in the similar crystalline states of **1** and **2** with different patterns of hydration/solvation and/or salts coin the term ‘pseudopolymorphism’ [29]. This phenomenon remains rare in supramolecular CD inclusion complexes. A few examples include two crystal forms of α-CD–camphor ethanol hydrate [30] and three forms of β-CD–EtOH hydrate [31]. Therefore, this study presents the first crystallographic evidence of CD inclusion complexes with drug/food molecules in freebase and HCl salt forms—β-CD–TRM base/HCl.

Crystal packing of the extraordinary α-CD–TRM HCl exclusion complex deserves further notes. Usually, the 3D arrangements of host CDs are induced by entire/partial inclusion of guests with varied sizes and shapes. To optimally encapsulate long guest molecules, CDs need to stack on top of each other, like coins in a row, yielding a channel-type structure [28]. However, for **3**, TRM does not fit into the α-CD cavity, but interacts with outer CD surface, forming an exclusion complex and facilitating the α-CD head-to-tail (H2T) column stacking along the *c*-axis (Figure 4c). Numerous crystal contacts stabilize the lattice of **3**, including various intermolecular O/C–H∙∙∙O/Cl H-bonds, particularly O6∙∙∙(O_w_)∙∙∙O2/O3 H-bonds at the H2T stack (Table S4).

To this point, what attracts our interest is the main difference between **1** and **2**, i.e., the round (**1**) and elliptical (**2**) β-CDs encapsulating the similar TRM molecules and the host–guest interactions contributing to the complex stabilization. Additionally, for **3**, is the partial inclusion complex of α-CD–TRM energetically plausible compared to the exclusion complex? These questions have been comprehensively addressed in Section 2.4 below.

### 2.4. Theoretical Viewpoints on the Distinct Associations of β- and α-CDs with TRM

**Figure 5:**
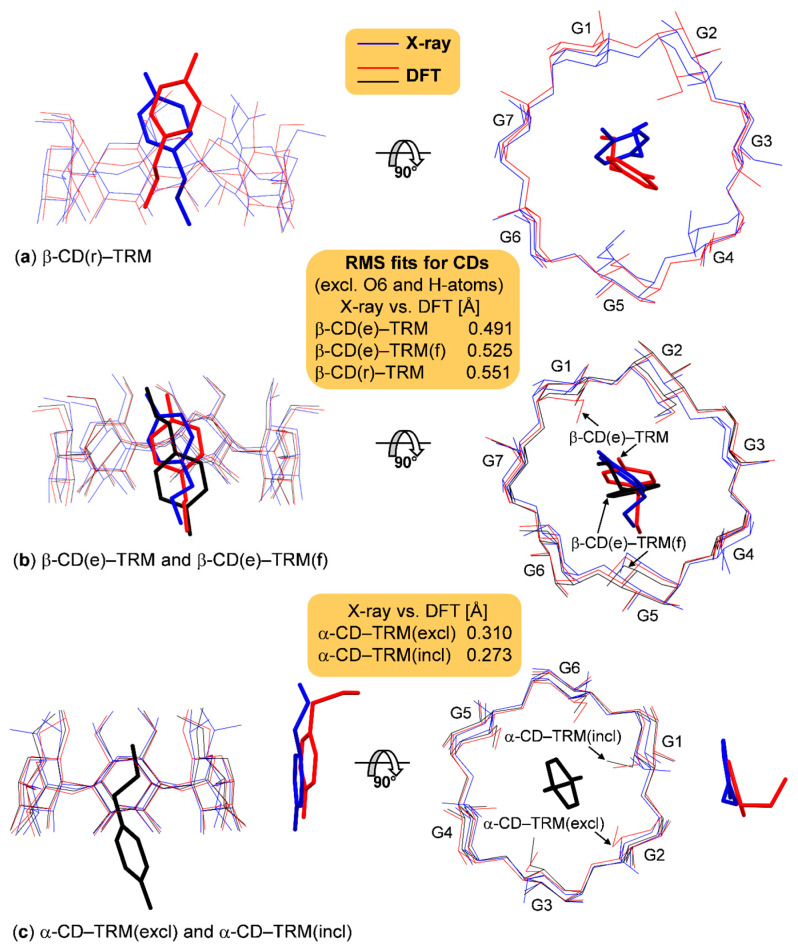
Structure superpositions of (**a**) β-CD(r)−TRM, (**b**) β-CD(e)−TRM and β-CD(e)−TRM(f), and (**c**) α-CD−TRM(excl) and α-CD−TRM(incl) complexes derived from X-ray analysis (blue lines) and DFT full-geometry optimization in the gas phase (red and black lines); see also Figure 6 and Tables S5–S7. The rms fits are calculated for non-H atoms of the CD skeletons, excluding TRM.

Single-crystal X-ray analysis combined with DFT full-geometry optimization provides a meaningful structure–energy correlation. Good starting atomic coordinates from X-ray analysis give a global energy-minimized structure in the gas phase that agrees quite well with the original solid-state structure; see for example, the CD–apple polyphenol inclusion complexes [6]. This is also evidenced from the moderate and small rms fits of the X-ray- and DFT-derived molecular structures of the respective β-CD (0.491–0.525 Å) and α-CD (0.273–0.310 Å) complexes; only non-H atoms of CDs are considered for the structure overlays (Figure 5). Concurrently, this emphasizes the reliability and validity of DFT calculation in the system of CD supramolecular complexes.

To reflect the meaning of X-ray-derived structures of **1** and **2**, the single point energy calculation was conducted on the hydrogen-distance-corrected X-ray structures. This is analogous to the interaction energy (Δ*E*_int_) from full-geometry optimization. The molecular structure of **1** with a round CD (β-CD(r)–TRM) has a slight positive Δ*E*_int_ of 0.89 kcal mol^−1^ (Table S7). This unexpected Δ*E*_int_ is due to the less accurately determined structure of **1**, i.e., three doubly disordered O6–H groups in β-CD and a half-occupied TRM in the β-CD cavity (see Section 2.2). In contrast, the molecular structure of **2** with an elliptical CD (β-CD(e)–TRM) has a normal Δ*E*_int_ of −4.57 kcal mol^−1^ (Table S7). Therefore, complete-geometry optimization is required for further insights, particularly the stabilization and deformation energies (Δ*E*_stb_ and Δ*E*_def_). As summarized in Table S7, Δ*E*_stb_s and Δ*E*_int_s of both β- and α-CD complexes fall in normal ranges of −6.96 to −13.01 and −7.92 to −16.98 kcal mol^−1^, indicating the nature of weak intermolecular noncovalent interactions in the CD supramolecular complexes.

**Figure 6:**
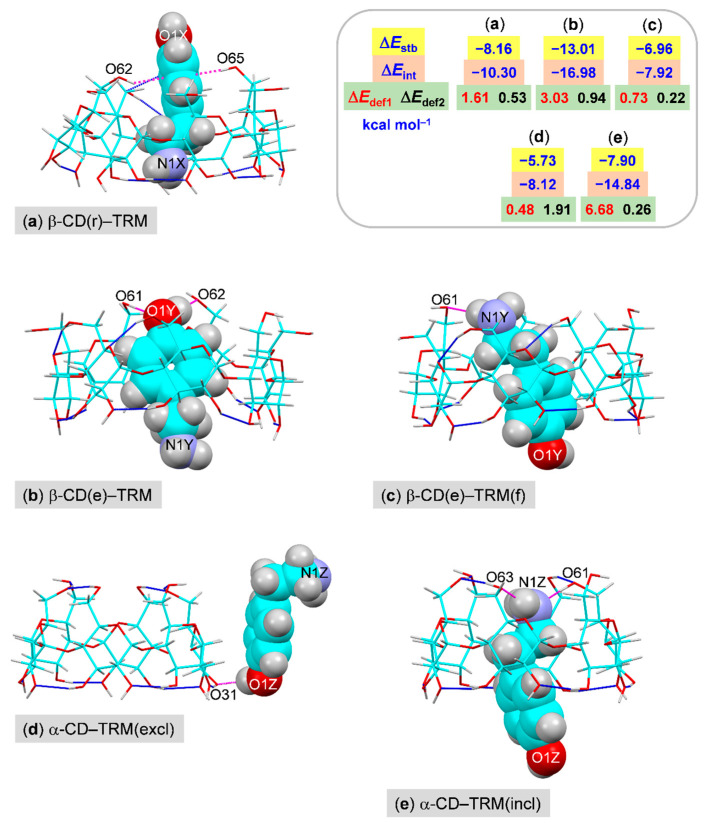
DFT-optimized structures of inclusion complexes (**a**) β-CD(r)−TRM, (**b**) β-CD(e)−TRM, (**c**) β-CD(e)−TRM(f), (**e**) α-CD−TRM(incl), and exclusion complex (**d**) α-CD−TRM(excl), shown only from side view; see also Figure 5 and Figure S1. The stabilization, interaction, and deformation energies (Δ*E*_stb_, Δ*E*_int_, Δ*E*_def1_, and Δ*E*_def2_) are summarized in the inset; see also Tables S5–S7. The intramolecular O–H∙∙∙O H-bonds within CDs and intermolecular N/O–H∙∙∙O/N H-bonds and O–H∙∙∙π interactions are indicated by blue and magenta connecting lines, respectively. The intramolecular, interglucose C6–H···O5 H-bonds and host–guest C–H···O/N interactions are not shown (Tables S5 and S6).

Expectedly, the β-CD(e)–TRM is more energetically stable than the β-CD(r)–TRM, as indicated by the relative energies ΔΔ*E*_stb_ and ΔΔ*E*_int_ of 4.85 and 6.68 kcal mol^−1^, respectively (Table S7). The β-CD(r)–TRM is stabilized by six O3···O2, one O6–H···O5, four C6–H···O5 H-bonds (within β-CD) and two O6–H···π interactions (intermolecular), while the β-CD(e)–TRM is stabilized by seven O3···O2, two O6–H···O5, three C6–H···O5 H-bonds (within β-CD), and two O61–H···O1Y–H···O62 and two C5–H···π interactions (intermolecular), Figure 6 and Figure S1 and Table 1 and Table S5. In both cases, the host–guest O/C–H···π interactions are established by the cooperative effects of the large inclination of two diametrically opposed glucoses G2 and G5 of β-CD (tilt angles ~30°), and the more-or-less vertical alignment of TRM in the CD cavity (Figure 6 and Figure S1 and Table 1 and Table S5).

A question remains: is the opposite inclusion mode thermodynamically plausible? To answer this question, the structure of the 180°-flipped TRM in the elliptical β-CD cavity (namely, β-CD(e)–TRM(f)) was built and its molecular energy was minimized. It turned out that β-CD(e)–TRM(f) is the least stable with the corresponding ΔΔ*E*_stb_ and ΔΔ*E*_int_ of 6.05 and 9.06 kcal mol^−1^ compared to the most stable β-CD(e)–TRM. The complex stabilization schemes of β-CD(e)–TRM(f) are intramolecularly similar to those of β-CD(e)–TRM but differ intermolecularly, as one N1Y–H1···O61 H-bond and two C–H···O/N interactions are formed only in the flipped mode. This is because glucose G5 is less tilted from 35.4° (X-ray) to 25.5° (DFT) for the optimal space-fit with the TRM aminoethyl side chain and the donation of H-bond from N1Y–H1 to O61 (Figure 6 and Figure S1 and Table 1 and Table S5). According to the induced-fit process [22], the β-CD(e)–TRM primarily gains thermodynamic stability through the structural changes of β-CD (greater) and TRM (lesser) with the respective deformation energies Δ*E*_def1_ and Δ*E*_def2_ of 3.03 and 0.94 kcal mol^−1^, which are 23% and 7% of Δ*E*_stb_, and ~2–4 times those of β-CD(r)–TRM and β-CD(e)–TRM(f) (Table S7). This is consistent with the deformation energies of inclusion complexes of drug piperazine with α-, β-, γ-CDs, and two β-CD methylated derivatives derived from the PM3 method [32].

Variation of the theme of α-CD–TRM complexation triggers curiosity. The occurrence and existence of the rare α-CD–TRM exclusion complex makes us astonished and recalls the two principles of CD inclusion complexation, viz., host–guest size compatibility and binding affinity. Hence, the original X-ray-derived α-CD–TRM exclusion complex and the corresponding inclusion complex built by 8.349-Å translation of TRM from outside to the α-CD centroid, respectively, namely, α-CD–TRM(excl) and α-CD–TRM(incl), are considered for DFT full-geometry optimization. Surprisingly, in vacuum (and solution), α-CD–TRM(incl) is more energetically stable than α-CD–TRM(excl) by 2.17 and 6.72 kcal mol^−1^, based on ΔΔ*E*_stb_ and ΔΔ*E*_int_, and intermolecular interactions of O61–H···N1Z–H2···O63 and O1Z–H···O31, C41–H···π···H–C12, respectively (Table 1, Tables S6 and S7). Comparing the DFT- to X-ray-derived structures, the right-angle aligned TRM moves its aromatic centroid horizontally (8.349 → 8.872 Å) and vertically (0.070 → 0.480 Å) from the α-CD molecular centroid, outside the cavity. For α-CD–TRM(incl), TRM aromatic ring makes an acute angle of 71.6° against and shifts 3.155 Å beneath the O4 plane, giving rise to the partial inclusion complex with the aminoethyl side chain (not the aromatic ring) enclosed in the α-CD cavity (Figure 5, Figure 6, and Figure S1). Such partial inclusions are frequently crystallographically observed for α-CD complexes with various guests bearing aromatic ring(s), e.g., *m*-nitroaniline [33], benzyl alcohol [34], and FEA [6]. Upon complexation, the six glucose units tilt further from 9.5–11.2° (X-ray, exclusion) to 14.6–17.8° (DFT, exclusion) and 11.0–17.8° (DFT, inclusion), facilitating intramolecular O3(*n*)–H···O2(*n* + 1), O6–H···O6, and C6–H···O5 H-bonds and maintaining the round α-CD macrocycles (Figure 5, Figure 6 and Figure S1 and Table 1 and Table S6). Exceptions are the two glucose units G2 and G6 of α-CD–TRM(incl) that are less inclined by 5.9° and 9.6° to open the O6–H side to accommodate the TRM aminoethyl moiety. In addition, the aminoethyl side chain in α-CD–TRM(excl) is oriented differently with the torsion angle C4–C7–C8–N1 of 62.4°, compared to those of other structures, ~180°. Consequently, α-CD and TRM molecules deform to more or less extent with Δ*E*_def1_, Δ*E*_def2_ of 6.68, 0.26 and 0.48, 1.91 kcal mol^−1^ for the respective inclusion and exclusion complexes (Figure 5, Figure 6 and Figure S1 and Table S7).

Furthermore, to improve the computational accuracy of DFT calculations, Δ*E*_int_s were reevaluated using the B97D/6-31+G(d,p) method and the BSSE correction [35]. After corrections, the stability-order retains together with Δ*E*_int_(B97D+BSSE)s (kcal mol^−1^): β-CD(e)–TRM (−29.18) > β-CD(r)–TRM (−24.00) > β-CD(e)–TRM(f) (−20.49); α-CD–TRM(incl) (−31.98) > α-CD–TRM(excl) (−12.26), Table S8. Note that a much greater contribution of dispersion forces to Δ*E*_int_s is due to the double surface contacts present in α-CD–TRM(incl) rather than the single surface contact in α-CD–TRM(excl). Importantly, when using larger basis sets, the effects of the basis set errors Δ*E*_BSSE_s on Δ*E*_int_(B97D)s significantly decrease to 11.9–15.3%, compared to ~25% in the β-CD–citalopram inclusion complexes [36]. The good structure–energy relationship established through this study of X-ray analysis combined with DFT calculation confirmed the genuine existence of CD–TRM supramolecular complexes and suggests the application of CDs in food safety: the electrochemical sensing of tyramine compounds using graphene–β-CD conjugates [37] and the reduction of the accumulation of tyramines in fermented sausages using the ferulic acid–hydroxypropyl-β-CD inclusion complexes [20]. Additionally, the pseudopolymorphs of β-CD–TRM base/HCl inclusion complexes exemplify the use of CDs in the formulation and delivery of drugs and bioactives [38].

## 3. Materials and Methods

### 3.1. Materials

α-CD (≥90%) and β-CD (≥95%) as fine white powdered solids were obtained from Cyclolab, Budapest, Hungary (codes CY-1001 and CY-2001). TRM freebase as brownish powders (>97%, code 14061) and TRM HCl as whitish powders (>99%, code 42238) were purchased from Acros Organics (now Thermo Scientific Chemicals), Geel, Belgium. Absolute EtOH (≥99.8%) was provided by the Liquor Distillery Organization, Excise Department, Chachoengsao, Thailand. All chemicals were used as received. Ultrapure water was supplied using a Milli-Q water system.

### 3.2. Single-Crystal Structure Determination

#### 3.2.1. Crystallization

The crystal growth method and condition of slow evaporation of 50% (*v*/*v*) EtOH–H_2_O solution of unimolar host–guest mixture were used here, as in the earlier studies on β-CD inclusion complexes with antidepressants [13] and food polyphenols [6]. Briefly, the relevant powders of 1:1 molar ratio of β-CD 50 mg (0.044 mmol), TRM base 6.0 mg, and TRM HCl 7.6 mg were mixed and readily dissolved in 1 mL of 50% (*v*/*v*) aqueous ethanol at 323 K, giving concentrated brown and colorless solutions of the inclusion complexes β-CD–TRM base (**1**) and β-CD–TRM HCl (**2**), respectively, both with a concentration of 0.044 M. After two weeks of evaporation in an air-conditioned room (298 K), prismatic single crystals of **1** and **2** were obtained. Note that the quantities of TRM base and TRM HCl used for crystallization corresponded to the water solubility of β-CD at 323 K, 52.7 mg mL^−1^ [39], significantly exceeding the solubility at 298 K, 18.5 mg mL^−1^. Additionally, both forms of TRM are already well-soluble in water at 288 K, HCl, 100 mg mL^−1^, and base, 10.4 mg mL^−1^, www.thermofisher.com (accessed on 14 May 2024). Hence, β-CD did not improve the water solubility of the TRM base/HCl. In contrast, the water solubility of ziprasidone mesylate (antipsychotic agent) was greatly enhanced by a factor of 10^5^, up to 40 mg mL^−1^, upon encapsulation of β-CD sulfobutyl ether, for intramuscular administration [40].

Crystallization attempts of the TRM HCl/base inclusion complex with the smaller α-CD ring (43 mg, 0.044 mmol) under identical conditions achieved partial success, despite the α- and β-CD complexes demonstrating comparative stability in solution [17,18]. While the brown solution of the α-CD–TRM base gave the crystals of free α-CD hydrate [24], the colorless solution of α-CD–TRM HCl provided the crystals of an unexpected exclusion complex (**3**).

#### 3.2.2. X-ray Diffraction Experiment

Several colorless single crystals of **1**–**3** were selected, and each was mounted on a microloop (MiTeGen, New York, NY, USA) and determined for unit cell dimensions and diffraction quality. While the plate-like crystals were **1**, the rod-like crystals were **2**. Both complexes belonged to the monoclinic space group *P*2_1_ with more or less similar unit cell parameters (see below). For **3**, the block-shaped crystals showed high symmetry in the orthorhombic space group *P*2_1_2_1_2 with unit cell constants as given below. The diffraction data of **1**–**3** were collected from the best diffracting crystals at 296 K to 0.83 Å atomic resolution on a Bruker PROSPECTOR CCD area-detector diffractometer (Bruker AXS, Karlsruhe, Germany) with an IµS microfocus X-ray source operating at 50 kV, 0.60 mA (CuKα radiation; λ = 1.54178 Å). Data processing typically proceeded in a sequential manner using the APEX2 software (version 2) suite [41]: (i) integration of diffraction image frames with SAINT [42], (ii) scaling and multi-scan absorption correction using SADABS [41], and (iii) data merging with XPREP [42]. This yielded good-quality diffraction data with coverage of 98.8–99.7% and *R*_int_ of 0.0223–0.0508, see the Supplementary Data, Table 2.

#### 3.2.3. Structure Solution and Refinement

The crystal structures of all complexes were solved using the intrinsic phasing method using SHELXTL XT [41]. In **1**, all non-H atoms of a round β-CD macrocycle, excluding three twofold disordered O6 and five and one isolated atoms were found outside and inside the CD cavity, respectively. Conversely, in **2,** all non-H atoms of a highly distorted round β-CD macrocycle with a twofold disordered O6, TRM, chloride and four isolated atoms outside the cavity were obtained. In **3**, a half of α-CD ring, a fragment of TRM and isolated atoms were found. For **1**, the remaining non-H atoms of β-CD O6, TRM, and some water sites were located through difference Fourier electron density maps. The atom types were rechecked and reassigned. Dozens of residual peaks inside the CD cavity were better modeled as a half-occupied TRM and a water cluster of 11 sites with occupancies of 0.2–0.6 rather than as only a twofold disordered TRM molecule. Seven water sites were found outside the CD cavity with occupancies of 0.2–1.0. For **2**, TRM, chloride, and four separate O atoms were freely refined to unity, i.e., these chemical constituents were well ordered. For **3**, TRM–H^+^ was in a special position of twofold-rotation symmetry passing through the midpoints of bonds C1–C2 and C4–C5 (Scheme 1), yielding a doubly disordered TRM–H^+^ with one-fourth occupancy factor. The resulting half-occupied TRM–H^+^ was counter-balanced by a half-occupied Cl^−^ in the intermolecular spaces. The water sites were clustered in the α-CD cavity and spread in the intermolecular interstices. Several CD O6 atoms with high thermal motions were suggested to be split, resulting in three, one, and six doubly disordered O6–H groups in **1**, **2,** and **3**, respectively. Anisotropic refinement using full-matrix least-squares on *F*^2^ with SHELXTL XLMP [41] was applied for all non-H atoms of **1**–**3**, except for the lowly occupied water sites, the half-occupied TRM base in the β-CD cavity (**1**), and the doubly disordered TRM–H^+^ outside the α-CD cavity, which were refined isotropically.

The H-atom positions were geometrically placed and handled with a riding model: C–H = 0.93 Å, *U*_iso_ = 1.2*U*_eq_(C)(aromatic); C–H = 0.98 Å, *U*_iso_ = 1.2*U*_eq_(C)(methine); C–H = 0.97 Å, *U*_iso_ = 1.2*U*_eq_(C)(methylene); C–H = 0.96 Å, *U*_iso_ = 1.5*U*_eq_(C)(methyl); N–H = 0.86 Å, *U*_iso_ = 1.5*U*_eq_(N)(1º-amine); and N–H = 0.89 Å, *U*_iso_ = 1.5*U*_eq_(N)(1º-ammonium). The hydroxyl H-atoms were positioned and refined using ‘AFIX 147’ or ‘AFIX 83’ with restraints O–H = 0.84 Å, *U*_iso_ = 1.5*U*_eq_(O). For **1** and **2**, the highly to fully occupied water sites, the H-atom positions were located through difference Fourier electron density maps and refined with DFIX restraints to an ideal geometry (O–H 0.96 Å and H···H 1.52 Å) and with ‘AFIX 3’ constraint *U*_iso_ = 1.5*U*_eq_(water). Those of the lowly occupied water sites could not be located. Although BUMP anti-bumping restraints were applied in the refinement course to prevent short intra- and intermolecular H⋅⋅⋅H distances, some H⋅⋅⋅H distances were somewhat short. Note that the water H-atoms were reliably determined, as evidenced by nearly all of them having H-bond acceptors. The refinements of **1** and **2** converged satisfactorily at rather low *R*_1_ values of 0.0551 and 0.0371, respectively. For **3**, the refinement was more difficult, as many restraints were used to maintain the TRM structure. TRM and water molecules were doubly and severely disordered with respective occupancy factors of 0.25 and 0.2–0.5; they were isotropically refined, giving rise to a higher final *R*_1_ of 0.0724.

Note that even though the 50% (*v*/*v*) EtOH–H_2_O solvent mixture was used for crystallization, ethanol was frequently not found to co-crystallize as it was not located through difference Fourier maps in the structure refinement stage. This was previously observed in the β-CD–antidepressant complexes [13] and again in **1**–**3**. Hence, the final chemical formulas of **1**–**3** were generally reliably determined through X-ray analysis. For further details, see Table 2.

### 3.3. DFT Complete-Geometry Optimization

X-ray crystallography and quantum calculations using the DFT method share the key results of structural chemistry research. X-ray analysis provides a space-and-time average structure over the crystal specimen and the diffraction data collection time, while DFT calculation gives a structure–physicochemical property relationship. Therefore, to establish a relevant structure–energy correlation, the total molecular energies are evaluated from the X-ray-derived structures. This methodology affords a global minimum energy structure without a negative vibrational frequency and expedites full-geometry optimization via the DFT method [6]. In previous DFT calculation of the β-CD–amitriptyline–H^+^ Cl^−^ complex [13], the coordinated Cl^−^ marginally affected the structure and stabilization energy of the complex. The β-CD–amitriptyline base complexes optimized in the gas phase and the SMD solvation model (water) were similar both in the structures and stabilization energies. In **2** and **3**, the disordered Cl^−^ ions were not linked to NH_3_^+^ but functioned as hydrogen bonding mediators analogous to hydration water molecules (see Section 2.2). Therefore, the β-CD–TRM base complexes with different starting atomic coordinates, including the X-ray-derived structures of the elliptical β-CD (two modes, mode 1, X-ray-derived orientation, and mode 2, 180º-flipped orientation) and the round β-CD (one mode), namely, β-CD(e)–TRM, β-CD(e)–TRM(f) and β-CD(r)–TRM were considered. Additionally, the α-CD exclusion and inclusion complexes with TRM base, viz., α-CD–TRM(excl) and α-CD–TRM(incl), respectively, derived and modified through X-ray analysis were also evaluated.

To improve the starting structure model from X-ray analysis, the underestimated C–H, N–H, and O–H distances (0.93–0.98, 0.86–0.89, and 0.82 Å) were, respectively, calibrated to the neutron H-distances (1.083, 1.009, and 0.983 Å) [43]. The normalized structures were first optimized using the semiempirical PM3 method and then fully re-optimized by DFT calculation in vacuum using the B3LYP functional with mixed basis sets 6-31+G(d) for H, N, O, and 4-31G for C for a compromise between computational cost and accuracy. This level of theory was successfully applied to the DFT energy optimization of the largest CD with 26 glucoses, cyclomaltohexaicosaose (CA-26) [44]. All calculations were conducted using GAUSSIAN09 [45]. The stabilization and interaction energies of the complex (Δ*E*_stb_ and Δ*E*_int_) were computed from the molecular energies of the relevant components using Equations (1) and (2). Upon inclusion complexation, the deformation energies of CD (Δ*E*_def1_) and TRM (Δ*E*_def2_) were also calculated using Equations (3)–(5).

Δ*E*_stb_ = *E*_cpx_ − (*E*_CD_opt_ + *E*_TRM_opt_) (1)

Δ*E*_int_ = *E*_cpx_ − (*E*_CD_sp_ + *E*_TRM_sp_) (2)

Δ*E*_def1_ = *E*_CD_sp_ − *E*_CD_opt_ (3)

Δ*E*_def2_ = *E*_TRM_sp_ − *E*_TRM_opt_ (4)

Δ*E*_stb_ = Δ*E*_int_ + Δ*E*_def1_ + Δ*E*_def2_ (5)

where *E*_cpx_, *E*_CD_opt_, and *E*_TRM_opt_ are the molecular energies from the full-geometry optimization of the complex, CD, and the TRM base, respectively, and *E*_CD_sp_ and *E*_TRM_sp_ are the corresponding single-point energies in the complexed states. For the optimized structures together with Δ*E*_stb_, Δ*E*_int_, see Section 2.4, Figure 5, Figure 6 and Figure S1, and Tables S6 and S7. The relevant intermolecular interactions underlying the complex stabilization of the DFT- and X-ray-derived structures are compared in Table 1.

Even while taking into account the dispersion interactions with the B97D functional and the basis set superposition error (BSSE) based on the counterpoise method [35], the B97D/6-31+G(d)/4-31G method gave overestimated Δ*E*_int_s by several ten percents due to the use of small basis sets, as demonstrated for the β-CD–antidepressant inclusion complexes [36,46]. The Δ*E*_int_ accuracy was therefore improved by using a larger basis set 6-31+G(d,p), Table S8.

## 4. Conclusions

Tyramine (TRM), a biogenic catecholamine neurotransmitter, is abundant in aged and fermented foods, processed meats, and alcoholic beverages and can trigger migraines and hypertension. TRM accumulated in foods is reduced and detected by using additive cyclodextrins (CDs), while their association characteristics remain ambiguous. To gain atomic-level understanding of CD–TRM complexation, single-crystal X-ray diffraction and density functional theory (DFT) calculation have been conducted, demonstrating the elusive pseudopolymorphs and the apparent induced-fit mechanism in CD inclusion complexes of β-CD·0.5TRM·7.6H_2_O (**1**) and β-CD·TRM HCl·4H_2_O (**2**), both in the monoclinic space group *P*2_1_, and the exclusion complex of α-CD·0.5(TRM HCl)·10H_2_O (**3**) in the orthorhombic space group *P*2_1_2_1_2.

Both **1** and **2** share the common inclusion mode with similar TRM structures in the distinct β-CD cavities: OH group near the O6–H rim and NH_2_/NH_3_^+^ group near the O2–H/O3–H rim. In **1**, the half-occupied TRM base places its aromatic centroid 2.455 Å above and inclines 68.8° against the O4 plane of the round β-CD, whereas in **2**, the well-ordered, protonated TRM–H^+^ is mostly vertically aligned (82.7°) in the elliptical β-CD cavity such that its aromatic centroid is 0.691 Å above the CD molecular plane. Both **1** and **2** have similar herringbone packing structures, which are mainly stabilized by intermolecular N/O–H∙∙∙O H-bonds. In **2**, β-CD tilts its two diametrically opposed glucoses, deforming to an elliptical shape to optimally anchor TRM–H^+^ and establish host–guest C–H∙∙∙π interactions, and the crystal is further stabilized by O–H∙∙∙Cl^–^ H-bonds. Furthermore, **3** differs from **2**, as the smaller α-CD ring prefers an exclusion complex with TRM HCl. The twofold symmetry-related, round α-CD cavity accommodates several disordered water sites instead. The twofold disordered, vertically aligned TRM sites are placed 0.070 Å above, 8.349 Å away from the α-CD molecular centroid and are primarily held in position by intermolecular O22–H_CD_⋯O1_TRM_ H-bond. In the lattice of orthorhombic, *P*2_1_2_1_2 (**3),** α-CDs are stacked in columns, forming a head-to-tail channel-type structure.

DFT calculations indicate that the X-ray-derived inclusion mode is more energetically favorable and the deformed β-CD to an elliptical shape contributes significantly to the complex thermodynamic stabilization. Furthermore, the inclusion complex of α-CD with the TRM aminoethyl side chain is also energetically favorable compared to the exclusion mode. The crystallographic evidence for the CD–TRM supramolecular complexes suggests the potential applications of CDs for food safety (detection and reduction of TRM) [20,37] and drug/bioactive formulation and delivery [38].

## Figures and Tables

**Table 1:** Intermolecular interactions stabilizing TRM base/HCl in complex with β- and α-CDs as derived from X-ray analysis and DFT calculation.

Interaction	D–H	H···A	D···A	∠(DHA)	Interaction	D–H	H···A	D···A	∠(DHA)
X-ray ^a^					DFT ^f^				
β-CD–TRM base (1)					β-CD(r)–TRM ^g^				
N1X–H1···O35 ^i b^	0.86	2.18	2.85(1)	134.6	O62–H···*Cg*1X ^c^	0.97	2.82	3.723	155.9
N1X–H2···O65A ^ii^	0.86	2.26	3.09(4)	160.6	O65–H···*Cg*1X	0.97	3.01	3.972	170.1
O1X–H···O1W ^iii^	0.82	2.13	2.89(2)	153.9					
O61–H···O1X ^iv^	0.82	1.95	2.76(2)	170.3					
C51–H···*Cg*1X ^c^	0.98	3.37	4.201(9)	143.6					
β-CD–TRM HCl (2)					β-CD(e)–TRM ^g^	0.82	2.05	2.803(5)	152.1
N1Y–H3···O61 ^v^	0.89	1.91	2.773(5)	163.0	O61–H···O1Y	0.98	1.94	2.908	172.2
N1Y–H1···O65 ^vi^	0.89	1.90	2.782(5)	174.0	O1Y–H···O62	0.99	1.83	2.788	162.0
N1Y–H2···O4W	0.89	1.91	2.804(5)	179.0	C52–H···*Cg*1Y ^d^	1.10	3.48	4.510	157.7
O1Y–H···O21 ^vii^	0.82	1.99	2.786(4)	164.7	C55–H···*Cg*1Y	1.10	3.77	4.723	146.4
C52–H···*Cg*1Y ^d^	0.98	3.21	4.133(4)	158.1	β-CD(e)–TRM(f) ^g^				
C56–H···*Cg*1Y	0.98	3.64	4.554(4)	156.8	N1Y–H1···O61	1.02	2.28	3.296	172.7
α-CD–TRM HCl (3)					α-CD–TRM(excl) ^g^				
O22–H···O1Z ^viii^	0.82	2.56	3.36(5)	168.9	O1Z–H···O31	0.98	1.95	2.877	156.7
C41–H···Cg1Z ^e^	0.98	2.765	3.728(4)	167.6	C41–H···Cg1Z ^e^	1.09	3.145	4.198	161.5
C61–H2···Cg1Z	0.97	4.022	4.730(8)	132.3	C12–H···Cg1Z	1.09	4.000	2.913	173.5
C12–H···Cg1Z	0.98	3.128	4.061(4)	150.5	α-CD–TRM(incl) ^g^				
					N1Z–H2···O63	1.03	2.11	3.109	165.3
					O61–H···N1Z	1.00	1.86	2.846	167.1

^a^ For a complete list of intermolecular interactions in **1**–**3**, see Tables S2–S4. ^b^ Equivalent positions: (i) −*x* + 1, *y* − 0.5, −*z* + 1; (ii) *x*, *y* − 1, *z*; (iii) −*x* + 1, *y* − 0.5, −*z* + 2; (iv) −*x* + 2, *y* − 0.5, −*z* + 2; (v) *x* + 1, *y*, *z*; (vi) −*x* + 2, *y* + 0.5, −*z* + 1; (vii) −*x* + 1, *y* − 0.5, −*z* + 1; (viii) −*x* + 1, −*y* + 1. ^c^ The aromatic plane of TRM in the round β-CD cavity: *Cg*1X = C1X–C2X–C3X–C4X–C5X–C6X. ^d^ The aromatic plane of TRM in the elliptical β-CD cavity: *Cg*1Y = C1Y–C2Y–C3Y–C4Y–C5Y–C6Y. ^e^ The aromatic plane of TRM in complex with α-CD: *Cg*1Z = C2Z–C3Z–C4Z–C4Z^vii^–C3Z^vii^–C2Z^vii^ (X-ray); C1Z–C2Z–C3Z–C4Z–C5Z–C6Z (DFT). ^f^ DFT energy minimization in vacuum at the B3LYP/6-31+G*/4-31G level; see also Tables S5–S7. ^g^ X-ray-derived structures were used as starting models; see also Figure S1 and Tables S5–S7: β-CD(r)–TRM—the round β-CD; β-CD(e)–TRM—the elliptical β-CD; β-CD(e)–TRM(f)—the elliptical β-CD embedding the 180°-flipped TRM; α-CD–TRM(excl)—the exclusion complex; and α-CD–TRM(incl)—the inclusion complex.

**Table 2:** X-ray single crystal data collection and refinement statistics of **1**–**3**.

	1	2	3
	β-CD–Tyramine base	β-CD–Tyramine HCl	α-CD–Tyramine HCl
CCDC number	2255519	2255520	2339039
Abbreviated formula	β-CD·0.5TRM·7.6H_2_O	β-CD·TRM·HCl·4H_2_O	α-CD·0.5(TRM·HCl)·10H_2_O
Chemical formula	(C_6_H_10_O_5_)_7_·0.5C_8_H_11_NO ·7.6H_2_O	(C_6_H_10_O_5_)_7_·C_8_H_11_NO·HCl ·4H_2_O	(C_6_H_10_O_5_)_6_·0.5(C_8_H_11_NO·HCl) ·10H_2_O
Formula weight	1330.81	1380.67	1217.13
Crystal habit, color	Plate, colorless	Rod, colorless	Block, colorless
Crystal size (mm)	0.06 × 0.18 × 0.20	0.20 × 0.22 × 0.28	0.32 × 0.34 × 0.50
Crystal system	Monoclinic	Monoclinic	Orthorhombic
Space group	*P*2_1_ (No. 4)	*P*2_1_ (No. 4)	*P*2_1_2_1_2 (No. 18)
*a*, *b*, *c* (Å)	15.0853(5), 10.3163(4), 20.8865(7)	12.5272(2), 18.5421(4), 14.0470(3)	16.6951(4), 21.9562(6), 8.2681(2)
*α*, *β*, *γ* (°)	90, 109.344(1), 90	90, 109.918(1), 90	90, 90, 90
*V* (Å^3^)	3067.0(2)	3067.7(1)	3030.8(1)
*Z*	2	2	2
*D_c_* (g cm^−3^)	1.441	1.495	1.334
*μ* (mm^−1^)	1.133	1.508	1.264
*F*(000)	1411	1468	1279
Diffractometer	PROSPECTOR Kappa CCD (Bruker)		
Operating power (kV, mA)	50, 0.600		
Wavelength (Å)	CuK_α_, 1.54178		
Temperature (K)	296(2)		
Crystal–detector dist. (mm)	40		
Data collection	*ω*–*φ* scan, 1.2° step, 3 s expose	*ω*–*φ* scan, 1.0° step, 6 s expose	*ω*–*φ* scan, 0.8° step, 4 s expose
Frames collected	2226	3030	1571
*Θ* range (°)	4.39–68.34	3.35–68.29	5.30–68.46
Resolution (Å)	0.83	0.83	0.83
Coverage (%), *R*_int_	98.8, 0.0454	99.7, 0.0508	99.4, 0.0223
Reflns collected/unique/observed	39,293/11,029/9080	42,676/11,053/9509	20,632/5523/5261
Data/parameters/restraints	11,029/841/27	11,053/854/15	5523/412/33
*R*_1_ ^a^, *wR*_2_ (*I* > 2*σ*(*I*)) ^b^	0.0551, 0.1532	0.0371, 0.0852	0.0724, 0.2078
*R*_1_, *wR*_2_ (all data), *S*	0.0649, 0.1640, 1.037	0.0471, 0.0904, 1.039	0.0746, 0.2132, 1.041
Δρ_min_, Δρ_max_ (e Å^−3^)	−0.24, 0.36	−0.28, 0.26	−0.48, 0.56
Flack parameter *x*	−0.06(8)	0.01(1)	0.41(2) ^c^

^a,b^ *R* = Σ||*F*_o_| − |*F*_c_||/Σ|*F*_o_|; *wR* = Σ{*w*(*F*_o_^2^– *F*_c_^2^)^2^/Σ*w*(*F*_o_^2^)^2^}^1/2^. ^c^ The absolute structure of **3** is reliably determined using the chiral reference α-CD molecule comprising six D-glucose units, although the *x* value deviates significantly from zero.

## Data Availability

The data are contained within the article. Additional crystallographic and computational data are available in the Supplementary Materials and the Cambridge Crystallographic Data Centre (CCDC).

## Supplementary Materials

The following supporting information can be downloaded at: https://www.mdpi.com/article/10.3390/ijms25147983/s1. Ref. [47] is cited in Supplementary Materials.
