# Supplementary material for: Variation of Cyclodextrin (CD) Complexation with Biogenic Amine Tyramine: Pseudopolymorphs of β-CD Inclusion vs. α-CD Exclusion, Deep Atomistic Insights
Source: Int J Mol Sci. 2024 Jul 22;25(14):7983. doi: 10.3390/ijms25147983 (PMC11277041; doi:10.3390/ijms25147983)
Supplement: Supplementary file 1 [file ijms-25-07983-s001.zip › 1_bcdtrm-fb_checkcif.pdf]

No syntax errors found.
Please wait while processing ....

CIF dictionary
Interpreting this report

Datablock: bcdtrm-fb_x6cu

Bond precision:	C-C = 0.0075 A		Wavelength=1.54178
Cell:	a=15.0853(5)	b=10.3163(4)	c=20.8865(7)
	alpha=90	beta=109.344(1)	gamma=90
Temperature:	296 K		

	Calculated	Reported
Volume	3066.95(19)	3066.95(19)
Space group	P 21	P 21
Hall group	P 2yb	P 2yb
Moiety formula	2(C42 H70 O35), C8 H11 N O, 2(O0.70), 0.6(O2), 5.6(H2 O), 7(O)	C42 H70 O35, C8 H11 N O, 2.8(H2 O), 4.8(O)
Sum formula	C92 H162.20 N O86.20	C46 H81.10 N0.50 O43.10
Mr	2661.63	1330.81
Dx,g cm-3	1.441	1.441
Z	1	2
Mu (mm-1)	1.133	1.133
F000	1410.8	1411.0
F000'	1416.60	
h,k,lmax	18,12,25	18,12,25
Nref	11256[5968]	11029
Tmin,Tmax	0.797,0.934	0.674,0.753
Tmin'	0.797	

Correction method= # Reported T Limits: Tmin=0.674 Tmax=0.753
AbsCorr = MULTI-SCAN

Data completeness= 1.85/0.98 Theta(max)= 68.336

R(reflections)= 0.0550(9080) wR2(reflections)= 0.1635(11029)

S = 1.040 Npar= 840

The following ALERTS were generated. Each ALERT has the format
test-name_ALERT_alert-type_alert-level.
Click on the hyperlinks for more details of the test.

Alert level A

```
PLAT417_ALERT_2_A Short Inter D-H..H-D       H1W3     ..H240     .       1.74 Ang.
                                                      x,y,z  =      1_555 Check
```

Author Response: Although BUMP restraints were used in the refinement course, some Inter H..H distances are somewhat short.

Alert level C

```
PLAT042_ALERT_1_C Calc. and Reported MoietyFormula Strings  Differ     Please Check
              Calc: 2(C42 H70 O35), C8 H11 N O, 2(O0.70), 0.6(O2), 5.6(H2 O), 7(
              Rep.: C42 H70 O35, C8 H11 N O, 2.8(H2 O), 4.8(O)
PLAT089_ALERT_3_C Poor Data / Parameter Ratio (Zmax < 18) ........       7.02 Note
PLAT202_ALERT_3_C Isotropic non-H Atoms in Anion/Solvent .........          2 Check
              O4WB     O5WA
PLAT241_ALERT_2_C High   'MainMol' Ueq as Compared to Neighbors of        C64 Check
PLAT242_ALERT_2_C Low    'MainMol' Ueq as Compared to Neighbors of        C54 Check
PLAT242_ALERT_2_C Low    'MainMol' Ueq as Compared to Neighbors of        C62 Check
PLAT260_ALERT_2_C Large Average Ueq of Residue Including       O1W      0.140 Check
PLAT340_ALERT_3_C Low Bond Precision on  C-C Bonds ...............    0.00747 Ang.
PLAT416_ALERT_2_C Short Intra D-H..H-D       H23O     ..H32O     .       1.92 Ang.
                                                      x,y,z  =      1_555 Check
PLAT416_ALERT_2_C Short Intra D-H..H-D       H26O     ..H35O     .       1.92 Ang.
                                                      x,y,z  =      1_555 Check
PLAT417_ALERT_2_C Short Inter D-H..H-D       H2W1     ..H22O     .       2.14 Ang.
                                              1-x,3/2+y,2-z  =      2_667 Check
```

Author Response: Although BUMP restraints were used in the refinement course, some Inter H..H distances are somewhat short.

```
PLAT911_ALERT_3_C Missing FCF Refl Between Thmin & STh/L=    0.600          20 Report
                2  0  0,    2  1  0, -16  5  2,  -2  0  2,  -1  0  2,   0  0  2,
               -2  0  3,    2  0  3, -18  0  6,  -9  0  6,   9  0  6,  -7 11  9,
```

```
                10  0 15, -11  5 21,  -9  6 21,  -1  6 21, -10  1 24,  -4  3 24,
                -1  0 24,  -5  0 25,
```

Alert level G

```
FORMU01_ALERT_1_G  There is a discrepancy between the atom counts in the
            _chemical_formula_sum and _chemical_formula_moiety. This is
            usually due to the moiety formula being in the wrong format.
            Atom count from _chemical_formula_sum:   C46 H81.1 N0.5 O43.1
            Atom count from _chemical_formula_moiety:C50 H86.6 N1 O43.6
PLAT002_ALERT_2_G Number of Distance or Angle Restraints on AtSite          3 Note
PLAT003_ALERT_2_G Number of Uiso or Uij Restrained non-H Atoms ...          4 Report
PLAT007_ALERT_5_G Number of Unrefined Donor-H Atoms ..............         33 Report
              H21O  H22O  H23O  H24O  H25O  H26O  H27O  H31O  H32O  H33O  H34O
              H35O  H36O  H37O  H61O  H62A  H63O  H64B  H65B  H66O  H67O  H62B
PLAT045_ALERT_1_G Calculated and Reported Z Differ by a Factor ...      0.500 Check
PLAT068_ALERT_1_G Reported F000 Differs from Calcd (or Missing)...     Please Check
PLAT171_ALERT_4_G The CIF-Embedded .res File Contains EADP Records          4 Report
PLAT172_ALERT_4_G The CIF-Embedded .res File Contains DFIX Records          1 Report
PLAT186_ALERT_4_G The CIF-Embedded .res File Contains ISOR Records          2 Report
PLAT230_ALERT_2_G Hirshfeld Test Diff for    O62B      --C62      .       5.6 s.u.
PLAT230_ALERT_2_G Hirshfeld Test Diff for    O64A      --C64      .       5.4 s.u.
PLAT300_ALERT_4_G Atom Site Occupancy of O1X        Constrained at        0.5 Check
```

And 38 other PLAT300 Alerts

More ...

```
PLAT301_ALERT_3_G Main Residue  Disorder .............(Resd    1)         5% Note
PLAT302_ALERT_4_G Anion/Solvent/Minor-Residue Disorder (Resd    2)       100% Note
```

And 13 other PLAT302 Alerts

More ...

```
PLAT304_ALERT_4_G Non-Integer Number of Atoms in ..... (Resd    2)      10.50 Check
```

And 13 other PLAT304 Alerts

More ...

```
PLAT311_ALERT_2_G Isolated Disordered Oxygen Atom (No H's ?) .....       O4WB Check
```

And 9 other PLAT311 Alerts

More ...

```
PLAT395_ALERT_2_G Deviating  X-O-Y   Angle From 120 for O13W     .      163.0 Degree
PLAT414_ALERT_2_G Short Intra D-H..H-X       H42      ..H62B     .       2.01 Ang.
                                                      x,y,z  =      1_555 Check
PLAT415_ALERT_2_G Short Inter D-H..H-X       H32O     ..H643     .       2.11 Ang.
                                                   x,-1+y,z  =      1_545 Check
PLAT417_ALERT_2_G Short Inter D-H..H-D       H23O     ..H64B     .       2.06 Ang.
                                                   x,-1+y,z  =      1_545 Check
```

Author Response: Although BUMP restraints were used in the refinement course, some Inter H..H distances are somewhat short.

```
PLAT417_ALERT_2_G Short Inter D-H..H-D       H32O     ..H64A     .       1.76 Ang.
                                                   x,-1+y,z  =      1_545 Check
PLAT432_ALERT_2_G Short Inter X...Y Contact  O31      ..C1X      .       2.97 Ang.
                                                   x,-1+y,z  =      1_545 Check
PLAT432_ALERT_2_G Short Inter X...Y Contact  O7W      ..C21      .       2.88 Ang.
                                                    x,1+y,z  =      1_565 Check
PLAT720_ALERT_4_G Number of Unusual/Non-Standard Labels ..........         19 Note
              H7X1    H7X2    H8X1    H8X2    H1X1    H1X2    H1W1    H2W1
              O2WA    H1W2    H2W2    O2WB    H1W3    H2W3    O4WA    O4WB
              O4WC    O5WA    O5WB
PLAT860_ALERT_3_G Number of Least-Squares Restraints .............         27 Note
PLAT883_ALERT_1_G No Info/Value for _atom_sites_solution_primary .     Please Do !
PLAT910_ALERT_3_G Missing # of FCF Reflection(s) Below Theta(Min).          3 Note
                  1  0  0,  -1  0  1,   0  0  1,
PLAT912_ALERT_4_G Missing # of FCF Reflections Above STh/L=  0.600         48 Note
PLAT933_ALERT_2_G Number of HKL-OMIT Records in Embedded .res File         13 Note
                  0  0  1,   1  0  0,   2  0  3,  -2  0  3,   0  0  2,  -1  0  1,
                 -2  0  2,  -9  0  6,   2  1  0,   2 -1  0,   9  0  6,   2  0  0,
                 -1  0  2,
PLAT969_ALERT_5_G The 'Henn et al.' R-Factor-gap value ..........       2.92 Note
              Predicted wR2: Based on SigI**2  5.59 or SHELX Weight 16.35
PLAT978_ALERT_2_G Number C-C Bonds with Positive Residual Density.          0 Info
PLAT992_ALERT_5_G Repd & Actual _reflns_number_gt Values Differ by          2 Check
```

```
  1 ALERT level A = Most likely a serious problem - resolve or explain
  0 ALERT level B = A potentially serious problem, consider carefully
 12 ALERT level C = Check. Ensure it is not caused by an omission or oversight
105 ALERT level G = General information/check it is not something unexpected

  5 ALERT type 1 CIF construction/syntax error, inconsistent or missing data
 31 ALERT type 2 Indicator that the structure model may be wrong or deficient
  7 ALERT type 3 Indicator that the structure quality may be low
```

```
  72 ALERT type 4 Improvement, methodology, query or suggestion
   3 ALERT type 5 Informative message, check
```

It is advisable to attempt to resolve as many as possible of the alerts in all categories. Often the minor alerts point to easily fixed oversights, errors and omissions in your CIF or refinement strategy, so attention to these fine details can be worthwhile. In order to resolve some of the more serious problems it may be necessary to carry out additional measurements or structure refinements. However, the purpose of your study may justify the reported deviations and the more serious of these should normally be commented upon in the discussion or experimental section of a paper or in the "special_details" fields of the CIF. checkCIF was carefully designed to identify outliers and unusual parameters, but every test has its limitations and alerts that are not important in a particular case may appear. Conversely, the absence of alerts does not guarantee there are no aspects of the results needing attention. It is up to the individual to critically assess their own results and, if necessary, seek expert advice.

Publication of your CIF in IUCr journals

A basic structural check has been run on your CIF. These basic checks will be run on all CIFs submitted for publication in IUCr journals (*Acta Crystallographica*, *Journal of Applied Crystallography*, *Journal of Synchrotron Radiation*); however, if you intend to submit to *Acta Crystallographica Section C* or *E* or *IUCrData*, you should make sure that full publication checks are run on the final version of your CIF prior to submission.

Publication of your CIF in other journals

Please refer to the *Notes for Authors* of the relevant journal for any special instructions relating to CIF submission.

PLATON version of 06/01/2024; check.def file version of 05/01/2024

Datablock bcdtrm-fb_x6cu - ellipsoid plot

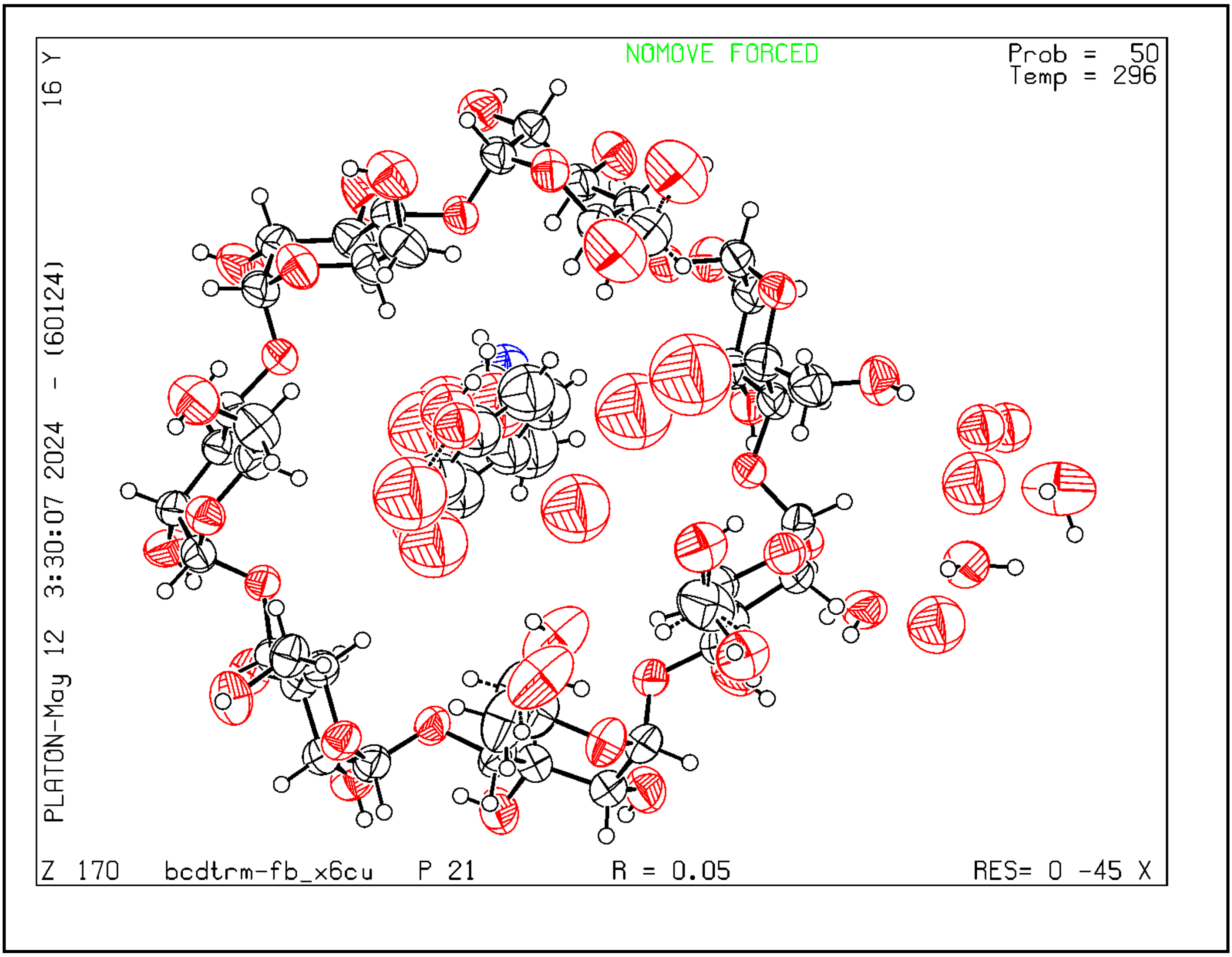

[Download CIF editor (publCIF) from the IUCr]
[Download CIF editor (enCIFer) from the CCDC]
[Test a new CIF entry]
