# Supplementary material for: Variation of Cyclodextrin (CD) Complexation with Biogenic Amine Tyramine: Pseudopolymorphs of β-CD Inclusion vs. α-CD Exclusion, Deep Atomistic Insights
Source: Int J Mol Sci. 2024 Jul 22;25(14):7983. doi: 10.3390/ijms25147983 (PMC11277041; doi:10.3390/ijms25147983)
Supplement: Supplementary file 1 [file ijms-25-07983-s001.zip › 2_bcdtrm-hcl_checkcif.pdf]

```
No syntax errors found.                         CIF dictionary
Please wait while processing ....               Interpreting this report
```

Datablock: bcdtrm-hcl_x6cu

Bond precision:	C-C = 0.0058 A		Wavelength=1.54178
Cell:	a=12.5272(2)	b=18.5421(4)	c=14.0470(3)
	alpha=90	beta=109.918(1)	gamma=90
Temperature: 296 K			

	Calculated	Reported
Volume	3067.67(11)	3067.67(11)
Space group	P 21	P 21
Hall group	P 2yb	P 2yb
Moiety formula	C42 H70 O35, C8 H12 N O, Cl, 4(H2 O)	C42 H70 O35, C8 H11 N O, H Cl, 4(H2 O)
Sum formula	C50 H90 Cl N O40	C50 H90 Cl N O40
Mr	1380.68	1380.67
Dx,g cm-3	1.495	1.495
Z	2	2
Mu (mm-1)	1.508	1.508
F000	1468.0	1468.0
F000'	1474.40	
h,k,lmax	15,22,16	15,22,16
Nref	11229[5804]	11053
Tmin,Tmax	0.897,0.913	0.604,0.753
Tmin'	0.617	

```
Correction method= # Reported T Limits: Tmin=0.604 Tmax=0.753
AbsCorr = MULTI-SCAN
Data completeness= 1.90/0.98        Theta(max)= 68.285
R(reflections)= 0.0371( 9509)            wR2(reflections)= 0.0903( 11053)
S = 1.038              Npar= 854
```

The following ALERTS were generated. Each ALERT has the format
test-name_ALERT_alert-type_alert-level.
Click on the hyperlinks for more details of the test.

Alert level B

```
PLAT417_ALERT_2_B Short Inter D-H..H-D       H2W2     ..H65O     .       2.09 Ang.
                                                      x,y,z  =      1_555 Check
```

Author Response: Although BUMP restraints were used in the refinement course, some Inter H..H distances are somewhat short.

```
PLAT420_ALERT_2_B D-H Bond Without Acceptor  O1W      --H1W1     .      Please Check
```

Author Response: Water H atoms were located from difference Fourier e- maps. After refinement, some H atoms moved and had no acceptor.

Alert level C

```
PLAT042_ALERT_1_C Calc. and Reported MoietyFormula Strings  Differ     Please Check
            Calc: C42 H70 O35, C8 H12 N O, Cl, 4(H2 O)
            Rep.: C42 H70 O35, C8 H11 N O, H Cl, 4(H2 O)
PLAT089_ALERT_3_C Poor Data / Parameter Ratio (Zmax < 18) ........       6.73 Note
PLAT260_ALERT_2_C Large Average Ueq of Residue Including       O1W      0.181 Check
PLAT340_ALERT_3_C Low Bond Precision on  C-C Bonds ..............    0.00584 Ang.
PLAT415_ALERT_2_C Short Inter D-H..H-X       H11      ..H1W4     .       2.05 Ang.
                                                   -1+x,y,z  =      1_455 Check
PLAT415_ALERT_2_C Short Inter D-H..H-X       H14      ..H66O     .       2.05 Ang.
                                                  1+x,y,1+z  =      1_656 Check
PLAT417_ALERT_2_C Short Inter D-H..H-D       H1W3     ..H66O     .       2.11 Ang.
                                                      x,y,z  =      1_555 Check
```

Author Response: Although BUMP restraints were used in the refinement course, some Inter H..H distances are somewhat short.

```
PLAT911_ALERT_3_C Missing FCF Refl Between Thmin & STh/L=    0.600          15 Report
               0  2  0,    7 19  1,    7 18  3,    3 21  3,    8 16  4,    2 21  4,
```

```
                 8 15  5,  10  0  8,   5  0 13,   5  1 13,  -2  9 13,   2  9 13,
                -8  0 14, -11  6 14,  -7  1 16,
```

Alert level G

```
PLAT007_ALERT_5_G Number of Unrefined Donor-H Atoms .............         34 Report
              H210  H220  H230  H240  H250  H260  H270  H310  H320  H330  H340
              H350  H360  H370  H610  H620  H630  H64B  H650  H660  H670  H64A
PLAT171_ALERT_4_G The CIF-Embedded .res File Contains EADP Records         1 Report
PLAT301_ALERT_3_G Main Residue  Disorder .............(Resd    1)         1% Note
PLAT414_ALERT_2_G Short Intra D-H..H-X       H632     ..H64A     .      2.09 Ang.
                                                      x,y,z  =      1_555 Check
PLAT415_ALERT_2_G Short Inter D-H..H-X       H360     ..H642     .      2.11 Ang.
                                              2-x,1/2+y,1-z  =      2_756 Check
PLAT417_ALERT_2_G Short Inter D-H..H-D       H270     ..H64B     .      1.80 Ang.
                                              2-x,1/2+y,1-z  =      2_756 Check
```

Author Response: Although BUMP restraints were used in the refinement course, some Inter H..H distances are somewhat short.

```
PLAT417_ALERT_2_G Short Inter D-H..H-D       H360     ..H64B     .      2.04 Ang.
                                              2-x,1/2+y,1-z  =      2_756 Check
PLAT720_ALERT_4_G Number of Unusual/Non-Standard Labels .........         15 Note
              H7Y1    H7Y2    H8Y1    H8Y2    H1Y1    H1Y2    H1Y3    H1W1
              H2W1    H1W2    H2W2    H1W3    H2W3    H1W4    H2W4
PLAT860_ALERT_3_G Number of Least-Squares Restraints ............         15 Note
PLAT883_ALERT_1_G No Info/Value for _atom_sites_solution_primary .    Please Do !
PLAT899_ALERT_4_G SHELXL2018 is Deprecated and Succeeded by SHELXL    2019/3 Note
PLAT912_ALERT_4_G Missing # of FCF Reflections Above STh/L=  0.600        40 Note
PLAT933_ALERT_2_G Number of HKL-OMIT Records in Embedded .res File         2 Note
                0  2  0,    0 -2  0,
PLAT969_ALERT_5_G The 'Henn et al.' R-Factor-gap value ..........       1.79 Note
              Predicted wR2: Based on SigI**2  5.04 or SHELX Weight  9.06
PLAT978_ALERT_2_G Number C-C Bonds with Positive Residual Density.         5 Info
```

```
   0 ALERT level A = Most likely a serious problem - resolve or explain
   2 ALERT level B = A potentially serious problem, consider carefully
   8 ALERT level C = Check. Ensure it is not caused by an omission or oversight
  15 ALERT level G = General information/check it is not something unexpected

   2 ALERT type 1 CIF construction/syntax error, inconsistent or missing data
  12 ALERT type 2 Indicator that the structure model may be wrong or deficient
   5 ALERT type 3 Indicator that the structure quality may be low
   4 ALERT type 4 Improvement, methodology, query or suggestion
   2 ALERT type 5 Informative message, check
```

It is advisable to attempt to resolve as many as possible of the alerts in all categories. Often the minor alerts point to easily fixed oversights, errors and omissions in your CIF or refinement strategy, so attention to these fine details can be worthwhile. In order to resolve some of the more serious problems it may be necessary to carry out additional measurements or structure refinements. However, the purpose of your study may justify the reported deviations and the more serious of these should normally be commented upon in the discussion or experimental section of a paper or in the "special_details" fields of the CIF. checkCIF was carefully designed to identify outliers and unusual parameters, but every test has its limitations and alerts that are not important in a particular case may appear. Conversely, the absence of alerts does not guarantee there are no aspects of the results needing attention. It is up to the individual to critically assess their own results and, if necessary, seek expert advice.

Publication of your CIF in IUCr journals

A basic structural check has been run on your CIF. These basic checks will be run on all CIFs submitted for publication in IUCr journals (*Acta Crystallographica*, *Journal of Applied Crystallography*, *Journal of Synchrotron Radiation*); however, if you intend to submit to *Acta Crystallographica Section C* or *E* or *IUCrData*, you should make sure that full publication checks are run on the final version of your CIF prior to submission.

Publication of your CIF in other journals

Please refer to the *Notes for Authors* of the relevant journal for any special instructions relating to CIF submission.

PLATON version of 06/01/2024; check.def file version of 05/01/2024

Datablock bcdtrm-hcl_x6cu - ellipsoid plot

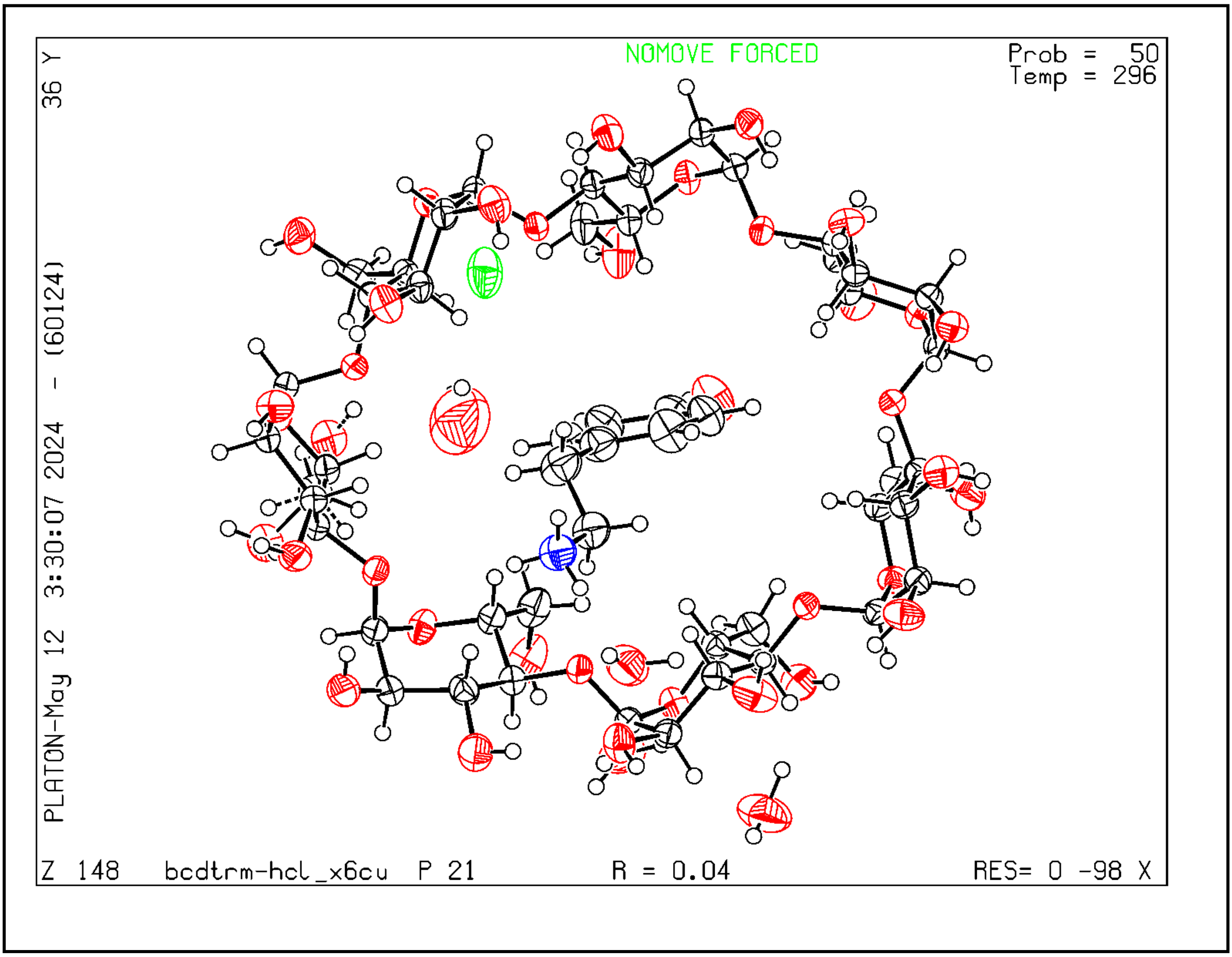

[Download CIF editor (publCIF) from the IUCr](#)
[Download CIF editor (enCIFer) from the CCDC](#)
[Test a new CIF entry](#)
