# Supplementary material for: Variation of Cyclodextrin (CD) Complexation with Biogenic Amine Tyramine: Pseudopolymorphs of β-CD Inclusion vs. α-CD Exclusion, Deep Atomistic Insights
Source: Int J Mol Sci. 2024 Jul 22;25(14):7983. doi: 10.3390/ijms25147983 (PMC11277041; doi:10.3390/ijms25147983)
Supplement: Supplementary file 1 [file ijms-25-07983-s001.zip › 3_acdtrm-hcl_checkcif.pdf]

No syntax errors found.
Please wait while processing ....

CIF dictionary
Interpreting this report

Datablock: acdtrm-hcl_b2x5

Bond precision:	C-C = 0.0063 A		Wavelength=1.54178
Cell:	a=16.6951(4)	b=21.9562(6)	c=8.2681(2)
	alpha=90	beta=90	gamma=90
Temperature:	296 K		

	Calculated	Reported
Volume	3030.76(13)	3030.76(13)
Space group	P 21 21 2	P 21 21 2
Hall group	P 2 2ab	P 2 2ab
Moiety formula	2(C36 H60 O30), 4(C2 H1.75 N0.25 O0.25), 6(O0.50), 2(O0.40), 2(	C36 H60 O30, 0.5(C8 H11 N O, H Cl), 10(H2 O)
Sum formula	C80 H127 Cl N O81	C40 H63.50 Cl0.50 N0.50 O40.50
Mr	2434.28	1217.13
Dx,g cm-3	1.334	1.334
Z	1	2
Mu (mm-1)	1.264	1.264
F000	1279.0	1279.0
F000'	1284.71	
h,k,lmax	20,26,9	20,26,9
Nref	5573[3166]	5523
Tmin,Tmax	0.627,0.667	0.589,0.753
Tmin'	0.506	

Correction method= # Reported T Limits: Tmin=0.589 Tmax=0.753
AbsCorr = MULTI-SCAN

Data completeness= 1.74/0.99 Theta(max)= 68.458

R(reflections)= 0.0724(5261) wR2(reflections)= 0.2132(5523)

S = 1.041 Npar= 412

The following ALERTS were generated. Each ALERT has the format
test-name_ALERT_alert-type_alert-level.
Click on the hyperlinks for more details of the test.

Alert level C

```
STRVA01_ALERT_4_C           Flack test results are ambiguous.
           From the CIF: _refine_ls_abs_structure_Flack    0.410
           From the CIF: _refine_ls_abs_structure_Flack_su    0.019
PLAT042_ALERT_1_C Calc. and Reported MoietyFormula Strings  Differ     Please Check
              Calc: 2(C36 H60 O30), 4(C2 H1.75 N0.25 O0.25), 6(O0.50), 2(O0.40),
              Rep.: C36 H60 O30, 0.5(C8 H11 N O, H Cl), 10(H2 O)
PLAT242_ALERT_2_C Low    'MainMol' Ueq as Compared to Neighbors of        C53 Check
PLAT260_ALERT_2_C Large Average Ueq of Residue Including        Cl1      0.119 Check
PLAT340_ALERT_3_C Low Bond Precision on  C-C Bonds ...............    0.00633 Ang.
PLAT430_ALERT_2_C Short Inter D...A Contact  O4WB      ..O51       .       2.88 Ang.
                                                 x,y,-1+z  =      1_554 Check
PLAT911_ALERT_3_C Missing FCF Refl Between Thmin & STh/L=    0.600         7 Report
               14  0  0,    2  1  0,    3  1  0,    5  3  0,    5  8  0,    0  0  1,
                1  2  4,
PLAT987_ALERT_1_C The Flack x is >> 0 -  Do a BASF/TWIN Refinement     Please Check
```

Alert level G

```
FORMU01_ALERT_1_G  There is a discrepancy between the atom counts in the
            _chemical_formula_sum and _chemical_formula_moiety. This is
            usually due to the moiety formula being in the wrong format.
            Atom count from _chemical_formula_sum:   C40 H63.5 Cl0.5 N0.5 O40.5
            Atom count from _chemical_formula_moiety:C40 H86.5 Cl1 N0.5 O40.5
PLAT002_ALERT_2_G Number of Distance or Angle Restraints on AtSite         16 Note
PLAT003_ALERT_2_G Number of Uiso or Uij Restrained non-H Atoms ...          6 Report
PLAT004_ALERT_5_G Polymeric Structure Found with Maximum Dimension          1 Info
PLAT007_ALERT_5_G Number of Unrefined Donor-H Atoms ..............         13 Report
              H21O  H22O  H23O  H31O  H32O  H33O  H61A  H62A  H63A  H61B  H62B
              H63B  H1Z
PLAT033_ALERT_4_G Flack x Value Deviates > 3.0 * sigma from Zero .      0.410 Note
PLAT045_ALERT_1_G Calculated and Reported Z Differ by a Factor ...      0.500 Check
PLAT072_ALERT_2_G SHELXL First  Parameter in WGHT  Unusually Large       0.17 Report
PLAT171_ALERT_4_G The CIF-Embedded .res File Contains EADP Records          3 Report
PLAT172_ALERT_4_G The CIF-Embedded .res File Contains DFIX Records         10 Report
```

```
PLAT186_ALERT_4_G The CIF-Embedded .res File Contains ISOR Records         1 Report
PLAT230_ALERT_2_G Hirshfeld Test Diff for    O62B     --C62      .        8.2 s.u.
PLAT300_ALERT_4_G Atom Site Occupancy of O1Z        Constrained at       0.25 Check
And 29 other PLAT300 Alerts
More ...
PLAT301_ALERT_3_G Main Residue  Disorder .............(Resd    1)         9% Note
PLAT302_ALERT_4_G Anion/Solvent/Minor-Residue Disorder (Resd    2)       100% Note
And 17 other PLAT302 Alerts
More ...
PLAT311_ALERT_2_G Isolated Disordered Oxygen Atom (No H's ?) .....       O2WA Check
And 13 other PLAT311 Alerts
More ...
PLAT415_ALERT_2_G Short Inter D-H..H-X       H31O     ..H613     .       2.14 Ang.
                                               x,y,-1+z  =      1_554 Check
PLAT417_ALERT_2_G Short Inter D-H..H-D       H22O     ..H1Z      .       2.11 Ang.
                                              1-x,1-y,z  =      2_665 Check
PLAT432_ALERT_2_G Short Inter X...Y Contact  Cl1      ..C7Z      .       2.65 Ang.
                                           1-x,1-y,-1+z  =      2_664 Check
PLAT432_ALERT_2_G Short Inter X...Y Contact  Cl1      ..C8Z      .       2.89 Ang.
                                           1-x,1-y,-1+z  =      2_664 Check
PLAT720_ALERT_4_G Number of Unusual/Non-Standard Labels .........          20 Note
              H7Z1    H7Z2    H8Z1    H8Z2    O1WA    O1WB    O2WA    O2WB
              O2WC    O3WA    O3WB    O4WA    O4WB    O4WC    O5WA    O5WB
              O5WC    O6WA    O6WB    O6WC
PLAT811_ALERT_5_G No ADDSYM Analysis: Too Many Excluded Atoms ....          ! Info
PLAT860_ALERT_3_G Number of Least-Squares Restraints ............          33 Note
PLAT883_ALERT_1_G No Info/Value for _atom_sites_solution_primary .     Please Do !
PLAT910_ALERT_3_G Missing # of FCF Reflection(s) Below Theta(Min).          3 Note
                1  1  0,    0  2  0,    1  2  0,
PLAT912_ALERT_4_G Missing # of FCF Reflections Above STh/L=  0.600          9 Note
PLAT933_ALERT_2_G Number of HKL-OMIT Records in Embedded .res File          6 Note
               14  0  0,  -1  2  4,    1  2  4,    5  8  0,    3  1  0,   2  1  0,
PLAT969_ALERT_5_G The 'Henn et al.' R-Factor-gap value ..........        8.07 Note
              Predicted wR2: Based on SigI**2  2.64 or SHELX Weight 21.28
PLAT978_ALERT_2_G Number C-C Bonds with Positive Residual Density.          1 Info
PLAT992_ALERT_5_G Repd & Actual _reflns_number_gt Values Differ by          4 Check
```

```
   0 ALERT level A = Most likely a serious problem - resolve or explain
   0 ALERT level B = A potentially serious problem, consider carefully
   8 ALERT level C = Check. Ensure it is not caused by an omission or oversight
  89 ALERT level G = General information/check it is not something unexpected

   5 ALERT type 1 CIF construction/syntax error, inconsistent or missing data
  27 ALERT type 2 Indicator that the structure model may be wrong or deficient
   5 ALERT type 3 Indicator that the structure quality may be low
  55 ALERT type 4 Improvement, methodology, query or suggestion
   5 ALERT type 5 Informative message, check
```

It is advisable to attempt to resolve as many as possible of the alerts in all categories. Often the minor alerts point to easily fixed oversights, errors and omissions in your CIF or refinement strategy, so attention to these fine details can be worthwhile. In order to resolve some of the more serious problems it may be necessary to carry out additional measurements or structure refinements. However, the purpose of your study may justify the reported deviations and the more serious of these should normally be commented upon in the discussion or experimental section of a paper or in the "special_details" fields of the CIF. checkCIF was carefully designed to identify outliers and unusual parameters, but every test has its limitations and alerts that are not important in a particular case may appear. Conversely, the absence of alerts does not guarantee there are no aspects of the results needing attention. It is up to the individual to critically assess their own results and, if necessary, seek expert advice.

Publication of your CIF in IUCr journals

A basic structural check has been run on your CIF. These basic checks will be run on all CIFs submitted for publication in IUCr journals (*Acta Crystallographica*, *Journal of Applied Crystallography*, *Journal of Synchrotron Radiation*); however, if you intend to submit to *Acta Crystallographica Section C* or *E* or *IUCrData*, you should make sure that full publication checks are run on the final version of your CIF prior to submission.

Publication of your CIF in other journals

Please refer to the *Notes for Authors* of the relevant journal for any special instructions relating to CIF submission.

PLATON version of 06/01/2024; check.def file version of 05/01/2024

Datablock acdtrm-hcl_b2x5 - ellipsoid plot

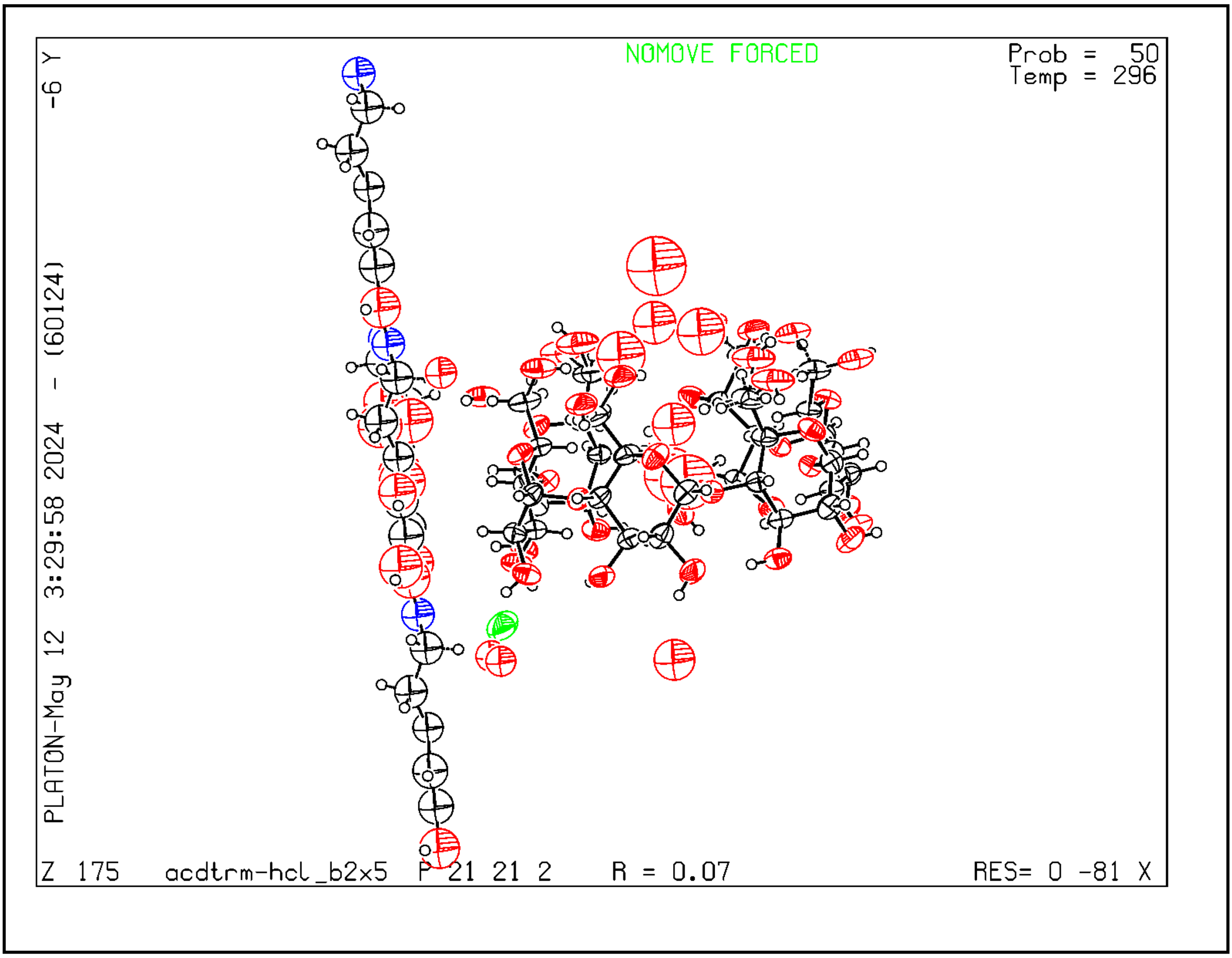

Download CIF editor (publCIF) from the IUCr
Download CIF editor (enCIFer) from the CCDC
Test a new CIF entry
