# Supplementary material for: Variation of Cyclodextrin (CD) Complexation with Biogenic Amine Tyramine: Pseudopolymorphs of β-CD Inclusion vs. α-CD Exclusion, Deep Atomistic Insights
Source: Int J Mol Sci. 2024 Jul 22;25(14):7983. doi: 10.3390/ijms25147983 (PMC11277041; doi:10.3390/ijms25147983)
Supplement: Supplementary file 1 [file ijms-25-07983-s001.zip › cdtrm_sm_rev21Jul24.pdf]

Supplementary Materials

Variation of Cyclodextrin (CD) Complexation with Biogenic Amine Tyramine: Pseudopolymorphs of β-CD Inclusion vs. α-CD Exclusion, Deep Atomistic Insights

Thammarat Aree

Department of Chemistry, Faculty of Science, Chulalongkorn University, Bangkok 10330, Thailand; Tel. +66-2-2187584; Fax +66-2-2187598; E-mail: athammar@chula.ac.th

https://orcid.org/0000-0002-7298-7401

Supplementary data available:

I. Crystallographic data

Table S1. Selected geometrical parameters of (**a**) β-CDs in β-CD·0.5TRM·7.6H_2O (**1**), β-CD·TRM·HCl·4H_2O (**2**) and β-CD·12H_2O (**i**). (**b**) α-CDs in α-CD·0.5(TRM·HCl)·10H_2O (**3**) and α-CD·6H_2O (**ii**).

Table S2. Intermolecular interactions stabilizing β-CD·0.5TRM·7.6H_2O (**1**).

Table S3. Intermolecular interactions stabilizing β-CD·TRM·HCl·4H_2O (**2**).

Table S4. Intermolecular interactions stabilizing α-CD·0.5(TRM·HCl)·10H_2O (**3**).

II. Computational data

Figure S1. DFT-optimized structures of inclusion complexes (**a**) β-CD(r)−TRM, (**b**) β-CD(e)−TRM, (**c**) β-CD(e)−TRM(f), (**e**) α-CD−TRM(incl), and exclusion complex (**d**) α-CD−TRM(excl).

Table S5. Hydrogen bond parameters and selected structural parameters of β-CD−TRM base inclusion complexes from different X-ray-derived starting atomic coordinates using DFT full-geometry optimization.

Table S6. Hydrogen bond parameters and selected structural parameters of α-CD−TRM base exclusion and inclusion complexes from different X-ray-derived starting atomic coordinates using DFT full-geometry optimization.

Table S7. Stabilization and interaction energies of β-CD and α-CD complex with TRM base from different X-ray-derived starting atomic coordinates using DFT full-geometry optimization and single-point energy calculation.

Table S8. Dispersion- and BSSE-corrected interaction energies of β-CD and α-CD complex with TRM base from DFT/B97D calculations.

III. References

I. Crystallographic data

Table S1. Selected geometrical parameters of (**a**) β-CDs in β-CD·0.5TRM·7.6H_2O (**1**), β-CD·TRM·HCl·4H_2O (**2**) and β-CD·12H_2O (**i**).

Residue	Puckering Q (Å) [a], θ (°) [b]			Tilt angle (°) [c]			O4 deviation (Å) [d]			O4(n)···O4(n – 1), O4(n)···centroid (Å)		
n	(**1**)	(**2**)	(**i**) [f]	(**1**)	(**2**)	(**i**) [f]	(**1**)	(**2**)	(**i**) [f]	(**1**)	(**2**)	(**i**) [f]
1	0.590(6)	0.572(4)	0.570	10.9(3)	10.0(2)	15.0	0.305(3)	–0.339(2)	0.192	4.456(4)	4.231(3)	4.489
	2.7(6)	5.7(4)	7.6							5.036(3)	4.990(2)	4.981
2	0.547(5)	0.577(4)	0.583	19.0(3)	23.5(1)	26.2	–0.168(3)	0.088(2)	0.091	4.222(4)	4.440(3)	4.392
	6.6(5)	3.8(4)	3.0							5.174(3)	4.439(2)	5.153
3	0.559(5)	0.571(4)	0.559	4.3(3)	8.1(1)	10.8	–0.186(3)	0.367(2)	–0.195	4.340(4)	4.499(3)	4.286
	1.6(5)	3.1(4)	3.9							4.895(3)	5.314(2)	5.122
4	0.566(7)	0.559(4)	0.596	17.7(3)	13.1(2)	7.9	0.229(3)	–0.290(2)	–0.053	4.486(5)	4.081(3)	4.443
	8.1(7)	10.6(4)	1.4							4.980(3)	5.350(2)	4.856
5	0.565(6)	0.557(4)	0.579	25.3(3)	35.4(2)	10.7	0.066(3)	–0.216(2)	0.276	4.361(4)	4.427(3)	4.452
	1.6(6)	6.4(4)	2.0							5.132(3)	4.568(2)	5.054
6	0.549(5)	0.583(4)	0.571	8.6(3)	13.8(1)	20.3	–0.217(2)	0.380(2)	–0.115	4.307(4)	4.635(3)	4.247
	3.9(5)	8.1(4)	3.9							5.113(3)	4.974(2)	5.184
7	0.561(5)	0.561(4)	0.567	9.1(1)	11.2(1)	6.4	–0.029(3)	0.011(2)	–0.196	4.408(4)	4.334(3)	4.338
	2.4(5)	3.7(4)	3.7							4.843(3)	5.467(2)	4.913
										0.264 [e]	*0.554*	*0.242*
										0.331 [e]	*1.028*	*0.328*
										0.870 [e]	*0.879*	*0.870*

[a,b] An ideal cyclohexane chair (for R(C–C) = 1.54 Å) has puckering amplitude Q = 0.63 Å and angle describing the polar position $\theta = 0°$ [1].
[c] Interplanar angle of the plane through C1(n), C4(n), O4(n) and O4(n – 1) against the O4 plane.
[d] Deviation of glycosidic O4 atoms from the least-squares plane through the seven O4 atoms.
[e] Ranges of the O4(n)···O4(n – 1), O4(n)···centroid distances and the average of their ratios are in *italics*, which are 0.868 for a perfect heptagon.
[f] β-CD·12H_2O [2], of which the glucose units are renumbered for the best fit to β-CD·0.5TRM·7.6H_2O (**1**).
[g] Endocyclic torsion angles ϕ and ψ at glycosidic O4, defined as O5(n + 1)–C1(n + 1)–O4(n)–C4(n) and C1(n + 1)–O4(n)–C4(n)–C5(n), respectively.
[h] Averages of ϕ and ψ are in *italics*; for the CD roundness, the sum of averages should be close to null.
[i] Exocyclic torsion angles χ and ω are defined as C4–C5–C6–O6 and O5–C5–C6–O6, respectively.
[j,k] Twofold disordered O62–H group of β-CD (**1**) with occupancy factors of 0.88 and 0.12 for sites A and B, respectively.
[l,m] Twofold disordered C64–H_2–O64–H group of β-CD (**1**) with occupancy factors of 0.30 and 0.70 for sites A and B, respectively.
[n,p] Twofold disordered O65–H group of β-CD (**1**) with occupancy factors of 0.21 and 0.79 for sites A and B, respectively.
[q,r] Twofold disordered O64–H group of β-CD (**2**) with an equal occupancy factor of 0.5 for both sites A and B.

Table S1. (**a**) Continued.

Residue	O3(*n*)···O2(*n* + 1) distance (Å)			Torsion angles ϕ [g], ψ [g] (°)			Torsion angles χ [i], ω [i] (°)		
n	(**1**)	(**2**)	(**i**) [f]	(**1**)	(**2**)	(**i**) [f]	(**1**)	(**2**)	(**i**) [f]
1	2.726(6)	2.750(4)	2.957	110.9(4)	110.0(3)	119.3	49.7(6)	176.7(3)	–169.4
				–113.9(5)	–116.9(3)	–95.9	–71.61(6)	55.7(4)	70.5
2	2.914(6)	2.768(4)	2.875	120.9(4)	120.7(3)	110.5	–179.2(6) [j] 64.0(32) [k]	175.8(4)	–173.9
				–112.5(4)	–105.0(3)	–106.6	59.4(7) [j] –57.3(32) [k]	59.8(5)	71.0
3	2.898(6)	2.970(4)	2.902	102.5(5)	103.6(3)	102.5	54.9(6)	53.5(4)	58.7
				–123.5(5)	–136.5(3)	–121.1	–65.6(5)	–65.0(3)	–60.8
4	2.921(6)	2.978(4)	2.783	115.9(5)	109.4(3)	107.7	–173.7(9) [l] 53.3(9) [m]	–176.3(4) [q] 33.8(5) [r]	57.0
				–97.5(6)	–103.6(3)	–109.4	66.3(11) [l] –66.7(9) [m]	63.2(4) [q] –86.6(5) [r]	–61.0
5	2.906(6)	2.930(4)	2.770	109.0(5)	113.3(3)	110.7	–163.2(35) [n] –177.1(8) [p]	64.3(4)	50.7
				–109.5(5)	–94.6(4)	–114.1	66.0(57) [n] 66.6(11) [p]	–55.53(4)	–71.0
6	2.899(5)	3.302(4)	2.855	102.1(5)	102.4(3)	120.0	59.4(5)	53.2(4)	–175.4
				–120.4(5)	–129.3(3)	–109.8	–60.9(5)	–66.2(4)	64.7
7	2.803(5)	2.853(4)	2.862	108.0(4)	97.3(3)	103.0	58.1(6)	60.1(4)	52.1
				–107.1(4)	–107.8(3)	–125.7	–62.1(6)	–61.6(4)	–62.9
				109.9 [h]	*108.1*	*110.5*			
				–112.1 [h]	*–113.4*	*–111.8*			
				–2.2 [h]	*–5.3*	*–1.3*			

[a,b] An ideal cyclohexane chair (for *R*(C–C) = 1.54 Å) has puckering amplitude *Q* = 0.63 Å and angle describing the polar position θ = 0° [1].
[c] Interplanar angle of the plane through C1(*n*), C4(*n*), O4(*n*) and O4(*n* – 1) against the O4 plane.
[d] Deviation of glycosidic O4 atoms from the least-squares plane through the seven O4 atoms.
[e] Ranges of the O4(*n*)···O4(*n* – 1), O4(*n*)···centroid distances and the average of their ratios are in *italics*, which are 0.868 for a perfect heptagon.
[f] β-CD·12H_2O [2], of which the glucose units are renumbered for the best fit to β-CD·0.5TRM·7.6H_2O (**1**).
[g] Endocyclic torsion angles ϕ and ψ at glycosidic O4, defined as O5(*n* + 1)–C1(*n* + 1)–O4(*n*)–C4(*n*) and C1(*n* + 1)–O4(*n*)–C4(*n*)–C5(*n*), respectively.
[h] Averages of ϕ and ψ are in *italics*; for the CD roundness, the sum of averages should be close to null.
[i] Exocyclic torsion angles χ and ω are defined as C4–C5–C6–O6 and O5–C5–C6–O6, respectively.
[j,k] Twofold disordered O62–H group of β-CD (**1**) with occupancy factors of 0.88 and 0.12 for sites A and B, respectively.
[l,m] Twofold disordered C64–H_2–O64–H group of β-CD (**1**) with occupancy factors of 0.30 and 0.70 for sites A and B, respectively.
[n,p] Twofold disordered O65–H group of β-CD (**1**) with occupancy factors of 0.21 and 0.79 for sites A and B, respectively.
[q,r] Twofold disordered O64–H group of β-CD (**2**) with an equal occupancy factor of 0.5 for both sites A and B.

Table S1. (**b**) α-CDs in α-CD·0.5(TRM·HCl)·10H_2O (**3**) and α-CD·6H_2O (**ii**).

Residue	Puckering *Q* (Å) [a], *θ* (º) [b]		Tilt angle (º) [c]		O4 deviation (Å) [d]		O4(*n*)···O4(*n* – 1), O4(*n*)···centroid (Å)		O3(*n*)···O2(*n* + 1) distance (Å)		Torsion angles *ϕ* [h], *ψ* [h] (º)		Torsion angles *χ* [j], *ω* [j] (º)	
n	(**3**) [f]	(**ii**) [g]	(**3**) [f]	(**ii**) [g]	(**3**) [f]	(**ii**) [g]	(**3**) [f]	(**ii**) [g]	(**3**) [f]	(**ii**) [g]	(**3**) [f]	(**ii**) [g]	(**3**) [f]	(**ii**) [g]
1	0.570(4)	0.575	11.2(1)	42.3	–0.016(2)	0.057	4.295(4)	4.433	2.839(5)	3.345	111.4(3)	90.6	179.6(4)[k] 50.0(12)[l]	–171.8
	0.0(4)	2.1					4.139(2)	4.010			–110.9(4)	–69.4	59.6(6)[k] –70.1(12)[l]	69.6
2	0.560(4)	0.593	9.5(1)	16.4	–0.001(2)	0.057	4.241(4)	4.405	2.855(5)	2.822	109.8(4)	88.2	–177.4(6)[m] 82.8(18)[n]	46.7
	4.7(5)	4.7					4.345(2)	4.299			–112.7(4)	–123.6	61.6(8)[m] –38.1(19)[n]	–74.1
3	0.556(5)	0.566	10.7(1)	12.1	0.018(2)	–0.135	4.204(4)	4.106	2.848(5)	3.015	111.3(4)	107.5	176.8(6)[p] 83.5(16)[q]	59.3
	6.2(5)	6.3					4.253(2)	4.471			–110.8(4)	–115.4	56.5(8)[p] –36.8(16)[q]	–63.3
4	0.570(4)	0.559	11.2(1)	9.4	–0.016(2)	0.092	4.295(4)	4.226	2.839(5)	2.940	111.4(3)	104.7	179.6(4)[k] 50.0(12)[l]	47.2
	0.0(4)	7.1					4.139(2)	4.087			–110.9(4)	–110.5	59.6(6)[k] –70.1(12)[l]	–71.8
5	0.560(4)	0.562	9.5(1)	22.5	–0.001(2)	0.023	4.241(4)	4.407	2.855(5)	3.026	109.8(4)	112.9	–177.4(6)[m] 82.8(18)[n]	–170.2
	4.7(5)	5.0					4.345(2)	4.214			–112.7(4)	–103.1	61.6(8)[m] –38.1(19)[n]	69.5
6	0.556(5)	0.548	10.7(1)	12.5	0.018(2)	–0.093	4.204(4)	4.282	2.848(5)	4.227	111.3(4)	100.5	176.8(6)[p] 83.5(16)[q]	52.4
	6.2(5)	6.5					4.253(2)	4.718			–110.8(4)	–118.5	56.5(8)[p] –36.8(16)[q]	–67.7
							0.091 [e]	*0.327*			*110.8* [i]	*100.7*		
							0.206 [e]	*0.708*			*–111.5* [i]	*–106.8*		
							1.001 [e]	*1.006*			*–0.6* [i]	*–6.0*		

[a,b] An ideal cyclohexane chair (for *R*(C–C) = 1.54 Å) has puckering amplitude *Q* = 0.63 Å and angle describing the polar position *θ* = 0º [1].

[c] Interplanar angle of the plane through C1(*n*), C4(*n*), O4(*n*) and O4(*n* – 1) against the O4 plane.

[d] Deviation of glycosidic O4 atoms from the least-squares plane through the six O4 atoms.

[e] Ranges of the O4(*n*)···O4(*n* – 1), O4(*n*)···centroid distances and the average of their ratios are in *italics*; for an ideal hexagon, the ratio is 1.000.

[f] α-CD·0.5(TRM·HCl)·10H_2O (**3**), whose glucose units G4, G5 and G6 are twofold symmetry generated G1, G2 and G3, respectively.

[g] α-CD·6H_2O [3], whose glucose units are rechecked for the best fit to α-CD·0.5(TRM·HCl)·10H_2O (**3**)

[h] Endocyclic torsion angles *ϕ* and *ψ* at glycosidic O4, defined as O5(*n* + 1)–C1(*n* + 1)–O4(*n*)–C4(*n*) and C1(*n* + 1)–O4(*n*)–C4(*n*)–C5(*n*), respectively.

[i] Averages of *ϕ* and *ψ* are in *italics*; for the CD roundness, the sum of averages should be close to null.

[j] Exocyclic torsion angles *χ* and *ω* are defined as C4–C5–C6–O6 and O5–C5–C6–O6, respectively.

[k,l] Twofold disordered O61–H (O64–H) group of α-CD (**3**) with occupancy factors of 0.81 and 0.19 for sites A and B, respectively.

[m,n] Twofold disordered O62–H (O65–H) group of α-CD (**3**) with occupancy factors of 0.87 and 0.13 for sites A and B, respectively.

[p,q] Twofold disordered O63–H (O66–H) group of α-CD (**3**) with occupancy factors of 0.84 and 0.16 for sites A and B, respectively.

Table S2. Intermolecular interactions stabilizing β-CD·0.5TRM·7.6H_2O (**1**) (Å, °).
(**a**) Hydrogen bond parameters (Å, °).

D–H···A	D–H	H···A	D···A	∠(DHA)	D–H···A	D–H	H···A	D···A	∠(DHA)
β-CD–β-CD/H_2O [a]					O2WA–H1···O23 [v]	0.96	1.92	2.736(6)	141.9
O21–H···O4WC [ii b]	0.82	2.18	2.97(2)	165.0	O2WA–H2···O37 [ix]	0.96	2.06	2.853(7)	139.1
O31–H···O22	0.82	1.94	2.726(6)	161.2	O3W–H1···O27 [iv]	0.96	2.13	2.737(5)	120.1
O61–H···O6W [iii]	0.82	1.99	2.77(2)	159.5	O3W–H2···O56 [vii]	0.96	2.07	2.925(5)	148.0
O22–H···O5WA [i]	0.82	1.96	2.71(3)	151.4	O3W–H2···O66 [vii]	0.96	2.49	3.153(7)	125.7
O22–H···O5WB [i]	0.82	2.25	2.92(4)	138.5	*TRM–β-CD/H_2O*				
O32–H···O23	0.82	2.35	2.914(6)	126.4	N1X–H1···O35 [vii]	0.86	2.18	2.85(1)	134.6
O32–H···O64A [i c]	0.82	2.08	2.57(2)	117.6	N1X–H2···O65A [i]	0.86	2.26	3.09(4)	160.6
O62A–H···O5WA	0.82	2.32	2.81(3)	119.2	O1X–H···O1W [xi]	0.82	2.13	2.89(2)	153.9
O23–H···O64B [i]	0.82	2.17	2.877(9)	143.6	O61–H···O1X [iii]	0.82	1.95	2.76(2)	170.3
O33–H···O24	0.82	2.12	2.898(6)	158.1	C51–H···*Cg*1X [d]	0.98	3.37	4.201(9)	143.6
O63–H···O4WB	0.82	2.04	2.854(8)	171.5	C54–H···*Cg*1X [e]	0.98	4.73	5.487(9)	137.1
O63–H···O4WC	0.82	2.1	2.81(2)	144.3	*H_2O–H_2O*				
O24–H···O3W	0.82	2.12	2.876(6)	154.1	O2WA–H1···O4WA	0.96	2.37	2.97(3)	119.8
O34–H···O36 [iv]	0.82	2.44	3.221(8)	159.5	C–H···O				
O64B–H···O14W [v]	0.82	2.60	3.01(2)	112.4	C31–H···O65B [i]	0.98	2.61	3.21(1)	119.1
O25–H···O13W [iv]	0.82	2.09	2.88(5)	160.3	C62–H1···O5WA	0.97	2.57	3.15(3)	118.2
O25–H···O14W [iv]	0.82	2.29	3.07(2)	159.2	C62–H2···O32 [xii]	0.97	2.65	3.399(8)	134.5
O35–H···O26	0.82	2.11	2.906(6)	162.8	C13–H···O4WA [i]	0.98	2.56	3.35(3)	137.7
O65A–H···O31 [v]	0.82	2.04	2.74(4)	143.5	C43–H···O57 [xiii]	0.98	2.57	3.528(5)	166.4
O65B–H···O13W [v]	0.82	2.31	2.77(6)	115.7	C63–H1···O62B [xii]	0.97	2.61	3.31(7)	129.2
O65B–H···O14W [v]	0.82	1.89	2.61(2)	145.9	C63–H2···O54	0.97	2.58	3.388(7)	141.4
O26–H···O33 [iv]	0.82	2.09	2.775(6)	141.0	C64–H1···O55	0.97	2.53	3.354(9)	142.4
O36–H···O3W [vi]	0.82	2.06	2.842(6)	160.0	C65B–H1···O56	0.97	2.59	3.41(2)	141.5
O66–H···O2WA [vi]	0.82	2.08	2.871(6)	163.4	C66–H1···O21 [v]	0.97	2.64	3.506(6)	149.4
O66–H···O2WB [vi]	0.82	2.05	2.80(4)	153.4	C66–H1···O37 [v]	0.97	2.59	3.415(6)	142.9
O27–H···O36	0.82	2.09	2.899(5)	167.2	C66–H2···O57	0.97	2.52	3.350(6)	142.9
O37–H···O21	0.82	2.05	2.803(5)	152.1	C17–H···O24 [vi]	0.98	2.50	3.468(6)	171.1
O67–H···O63 [vi]	0.82	2.02	2.817(7)	164.9	C27–H···O33 [vi]	0.98	2.58	3.391(6)	140.2
O1W–H1···O67 [ix]	0.96	1.78	2.715(9)	165.0	C2X–H2···O1W [xi]	0.93	2.47	3.16(1)	131.7
O1W–H2···O22 [x]	0.96	1.84	2.789(7)	169.0					

[a] Site occupancy factors (SOFs) are as follows:
4.3 and 3.3 water molecules are distributed over 7 and 11 sites outside the cavity and inside/near the cavity, respectively:
7 sites with SOFs = 1.0 (O1W, O3W); 0.8 (O2WA); 0.7 (O4WB); 0.3 (O4WA/C) and 0.2 (O2WB).
11 sites with SOFs = 0.6 (O5WA); 0.4 (O5WB, O8W); 0.3 (O6W, O7W, O14W); and 0.2 (O9W–O13W).
TRM was refined to a half occupancy inside the CD cavity.

[b] Equivalent positions: (i) *x*, *y* – 1, *z*; (ii) *x* + 1, *y* – 1, *z*; (iii) –*x* + 2, *y* – 0.5, –*z* + 2; (iv) –*x* + 1, *y* + 0.5, –*z* + 1; (v) *x*, *y* + 1, *z*; (vi) *x* + 1, *y*, *z*; (vii) –*x* + 1, *y* – 0.5, –*z* + 1; (viii) –*x* + 2, *y* + 0.5, –*z* + 2; (ix) *x* – 1, *y* + 1, *z*; (x) –*x* + 1, *y* + 1.5, –*z* + 2; (xi) –*x* + 1, *y* – 0.5, –*z* + 2; (xii) –*x* + 1, *y* + 0.5, –*z* + 2; (xiii) *x* – 1, *y*, *z*.

[c] Twofold disordered O62–H group of β-CD (**1**) with occupancy factors of 0.88 and 0.12 for sites A and B, respectively.
Twofold disordered O64–H group of β-CD (**1**) with occupancy factors of 0.30 and 0.70 for sites A and B, respectively.
Twofold disordered O65–H group of β-CD (**1**) with occupancy factors of 0.21 and 0.79 for sites A and B, respectively.

[d] The aromatic plane of TRM: *Cg*1X = C1X–C2X–C3X–C4X–C5X–C6X.

[e] The C54–H···*Cg*1X interaction is negligible due to the too long H···*Cg*1X distance.

(**b**) Intermolecular distances involving water sites (Å). [a]

D–H···A	D–H	H···A	D···A	∠(DHA)	D–H···A	D–H	H···A	D···A	∠(DHA)
O2WB···O23 [i,b]			2.55(4)		O6W···O51 [vi]			3.16(2)	
O2WB···O37 [ii]			3.12(5)		O6W···O61 [vi]			2.78(2)	
O2WB···O66 [iii]			2.81(4)		O7W···O21 [i]			2.57(5)	
O4WA···O2WA			2.97(3)		O7W···O31 [i]			2.71(5)	
O4WA···O1W			2.82(3)		O7W···O8W			2.07(5)	
O4WA···O23 [i]			3.20(3)		O8W···O21 [vi]			2.57(4)	
O4WA···O4WC			2.72(4)		O8W···O31 [i]			3.03(3)	
O4WA···O62A [iv,c]			2.16(3)		O8W···O9W [i]			3.11(3)	
O4WB···O2WA			2.83(1)		O8W···O9W			2.94(7)	
O4WB···O63			2.854(9)		O8W···O10W			3.05(6)	
O4WB···O52 [iv]			3.143(8)		O8W···O11W			3.00(6)	
O4WB···O61 [iv]			2.882(9)		O8W···O65A			3.02(6)	
O4WB···O62A [iv]			2.98(1)		O9W···O10W			2.54(8)	
O4WC···O2WA			2.77(2)		O9W···O64A			2.64(7)	
O4WC···O21 [ii]			2.98(2)		O9W···O65A			2.76(8)	
O4WC···O63			2.81(2)		O10W···O12W			2.81(8)	
O4WC···O61 [iv]			2.61(2)		O11W···O12W			1.82(8)	
O5WA···O22 [i]			2.71(3)		O11W···O13W			2.68(8)	
O5WA···O62A			2.81(3)		O11W···O25 [vii]			2.84(4)	
O5WA···O62B [iv]			2.78(8)		O13W···O25 [vii]			2.88(6)	
O5WB···O22 [i]			2.92(4)		O13W···O65B [viii]			2.77(6)	
O5WB···O9W			2.29(7)		O14W···O64B [viii]			3.01(2)	
O5WB···O10W			2.80(7)		O14W···O25 [vii]			3.07(2)	
O5WB···O64A			2.94(4)		O14W···O35 [vii]			2.68(2)	
O6W···O31 [i]			2.81(2)		O14W···O55 [viii]			3.12(1)	
O6W···O8W			3.23(3)		O14W···O65A [viii]			3.03(5)	
O6W···O10W			3.22(5)		O14W···O65B [viii]			2.61(2)	
O6W···O1W [v]			2.82(2)						

[a] Site occupancy factors (SOFs) are as follows:
4.3 and 3.3 water molecules are distributed over 7 and 11 sites outside the cavity and inside/near the cavity, respectively:
7 sites with SOFs = 1.0 (O1W, O3W); 0.8 (O2WA); 0.7 (O4WB); 0.3 (O4WA/C) and 0.2 (O2WB).
11 sites with SOFs = 0.6 (O5WA); 0.4 (O5WB, O8W); 0.3 (O6W, O7W, O14W); and 0.2 (O9W–O13W).
[b] Equivalent positions: (i) *x*, 1 + *y*, *z*; (ii) –1 + *x*,1 + *y*, *z*; (iii) –1 + *x*, *y*, *z*; (iv) 1 – *x*, 0.5 + *y*, 2 – *z*;
(v) 1 – *x*, –0.5 + *y*, 2 – *z*; (vi) 2 – *x*, 0.5 + *y*, 2 – *z*; (vii) 1 – *x*, –0.5 + *y*, 1 – *z*; (viii) *x*, –1 + *y*, *z*.
[c] Twofold disordered O62–H group of β-CD (**1**) with occupancy factors of 0.88 and 0.12 for sites A and B, respectively.
Twofold disordered O64–H group of β-CD (**1**) with occupancy factors of 0.30 and 0.70 for sites A and B, respectively.
Twofold disordered O65–H group of β-CD (**1**) with occupancy factors of 0.21 and 0.79 for sites A and B, respectively.

Table S3. Intermolecular interactions stabilizing β-CD·TRM·HCl·4H_2O (**2**) (Å, °).

D–H···A	D–H	H···A	D···A	∠(DHA)	D–H···A	D–H	H···A	D···A	∠(DHA)
β-CD–β-CD/H_2O/Cl^- [a]					O37–H···Cl1 ^{vii}	0.82	2.36	3.171(3)	171.0
O21–H···O37	0.82	2.12	2.853(4)	148.9	O67–H···O53 ^{vi}	0.82	2.64	3.101(4)	117.2
O31–H···O25 ^{v}	0.82	1.91	2.668(4)	153.1	O67–H···O63 ^{vi}	0.82	2.05	2.854(4)	168.3
O61–H···O34 ^{viii}	0.82	2.07	2.764(4)	142.5	*TRM–H^+–β-CD/H_2O*				
O22–H···O31	0.82	1.97	2.750(4)	158.8	N1Y–H3···O61 ^{iii}	0.89	1.91	2.773(5)	163.0
O32–H···O23	0.82	1.99	2.768(4)	159.3	N1Y–H1···O65 ^{v}	0.89	1.90	2.782(5)	174.0
O62–H···Cl1	0.82	2.39	3.154(3)	155.7	N1Y–H2···O4W	0.89	1.91	2.804(5)	179.0
O33–H···O24	0.82	2.15	2.970(4)	173.1	O1Y–H···O21 ^{ix}	0.82	1.99	2.786(4)	164.7
O63–H···O22 ^{i} [b]	0.82	2.32	3.030(4)	146.1	C52–H···*Cg*1Y [d]	0.98	3.21	4.133(4)	158.1
O63–H···O32 ^{i}	0.82	2.39	3.044(4)	137.4	C56–H···*Cg*1Y [d]	0.98	3.64	4.554(4)	156.8
O24–H···O52 ^{iii}	0.82	2.44	3.132(4)	142.9	*H_2O–H_2O/Cl^-*				
O24–H···O62 ^{iii}	0.82	2.44	3.156(5)	147.1	O1W–H2···Cl1	0.96	2.56	3.303(8)	134.6
O34–H···O62 ^{iii}	0.82	2.05	2.868(4)	177.6	O2W–H1···O3W	0.96	2.42	3.071(6)	124.6
O64A–H···O1W	0.82	2.17	2.595(9)	112.0	O3W–H2···Cl1 ^{iv}	0.96	2.23	3.146(5)	160.0
O4W–H2···O64A ^{v} [c]	0.96	1.97	2.877(7)	156.9	O4W–H1···O1W ^{v}	0.96	2.50	3.176(11)	127.1
O64B–H···O27 ^{ii}	0.82	1.81	2.598(6)	159.3	C–H···O				
O25–H···O34	0.82	2.26	2.978(4)	147.1	C42–H···O36 ^{x}	0.98	2.47	3.416(5)	161.2
O35–H···O26	0.82	2.13	2.930(4)	164.5	C43–H···O57 ^{xi}	0.98	2.65	3.603(4)	163.7
O65–H···O2W	0.82	2.05	2.630(5)	126.9	C63–H2···O54	0.97	2.55	3.319(5)	135.9
O26–H···O53 ^{iv}	0.82	2.04	2.835(4)	162.3	C14–H···O66 ^{xi}	0.98	2.62	3.435(4)	141.1
O36–H···O64B ^{v}	0.82	1.96	2.774(6)	171.8	C44–H···O37 ^{ii}	0.98	2.54	3.451(4)	155.3
O2W–H1···O56	0.96	2.09	2.838(4)	133.4	C64–H1···O55	0.97	2.54	3.420(5)	150.4
O66–H···O57	0.82	2.58	3.162(4)	129.4	C64–H2···O27 ^{ii}	0.97	2.64	3.573(5)	161.2
O3W–H1···O66	0.96	1.76	2.700(5)	166.4	C15–H···O64B	0.98	2.66	3.312(7)	124.3
O27–H···O36	0.82	2.55	3.303(5)	153.9	C66–H2···O57	0.97	2.56	3.183(5)	122.0
					C17–H···O24 ^{vi}	0.98	2.58	3.540(5)	166.5

[a] Site occupancy factors (SOFs) are as follows:
Four water molecules (O1W–O4W) are well ordered in the intermolecular interstices.
One TRM–H^+ and one chloride, each was refined to unity.

[b] Equivalent positions: (i) $-x + 2, y - 0.5, -z + 2$; (ii) $-x + 2, y - 0.5, -z + 1$; (iii) $x + 1, y, z$; (iv) $x, y, z - 1$; (v) $-x + 2, y + 0.5, -z + 1$; (vi) $x - 1, y, z - 1$; (vii) $-x + 1, y + 0.5, -z + 1$; (viii) $x - 1, y, z$; (ix) $-x + 1, y - 0.5, -z + 1$; (x) $x, y, z + 1$; (xi) $x + 1, y, z + 1$.

[c] Twofold disordered O63–H group of β-CD (**2**) with an equal occupancy factor of 0.5 for both sites A and B.

[d] The aromatic plane of TRM–H^+: *Cg*1Y = C1Y–C2Y–C3Y–C4Y–C5Y–C6Y.

Table S4. Intermolecular interactions stabilizing α-CD·0.5(TRM·HCl)·10H_2O (**3**) (Å, °).
(**a**) Hydrogen bond parameters (Å, °).

D–H···A	D–H	H···A	D···A	∠(DHA)	D–H···A	D–H	H···A	D···A	∠(DHA)
α-CD–α-CD/H_2O/Cl^- [a]					*TRM^+–α-CD/H_2O*				
O21–H···O4WA	0.82	2.05	2.752(12)	144.0	O22–H···O1Z ^{ii}	0.82	2.56	3.36(5)	168.9
O21–H···O4WB	0.82	2.08	2.728(11)	135.3	C41–H···*Cg*1Z [d]	0.98	2.765	3.728(4)	167.6
O21–H···O4WC ^{iv}	0.82	2.30	3.03(3)	148.2	C61–H2···*Cg*1Z [d]	0.97	4.022	4.730(8)	132.3
O31–H···O61A ^{iv}	0.82	1.94	2.758(6)	174.7	C12–H···*Cg*1Z [d]	0.98	3.128	4.061(4)	150.5
O61B–H[c]···O1WA ^{ii}	0.82	1.92	2.46(5)	123.3	C–H···O/Cl^-				
O22–H···O2WC ^{ii}	0.82	2.31	3.12(5)	169.1	C11–H···O32 ^{iii}	0.98	2.59	3.554(5)	169.0
O32–H···O23 ^{i} [b]	0.82	2.07	2.855(5)	159.4	C61–H1···O52	0.97	2.59	3.411(6)	142.2
O62A–H···O32 ^{v}	0.82	2.19	2.732(6)	124.2	C61–H4···O5WB	0.97	2.63	3.26(5)	122.6
O62A–H···Cl1 ^{v}	0.82	2.67	3.180(11)	121.4	C12–H···O2WB ^{ii}	0.98	2.62	3.57(3)	161.1
O62B–H···O4WB ^{viii}	0.82	2.15	2.72(4)	126.0	C62–H4···O53	0.97	2.51	3.442(8)	160.4
O23–H···O3WA	0.82	2.40	3.06(4)	138.6	C63–H4···O51 ^{i}	0.97	2.51	3.441(7)	160.0
O23–H···O3WB	0.82	2.00	2.716(9)	145.4	C63–H2···Cl1 ^{vi}	0.97	2.93	3.785(15)	148.3
O33–H···O21	0.82	2.18	2.848(5)	138.7	C63–H4···O51 ^{i}	0.97	2.51	3.441(7)	160.0
O63A–H···O5WA	0.82	2.02	2.76(4)	148.6	C7T–H1···Cl1 ^{vii}	0.97	2.21	2.64(3)	105.7
O63B–H···Cl1 ^{vi}	0.82	2.01	2.66(3)	135.8	C8T–H1···Cl1 ^{vii}	0.97	2.40	2.89(3)	110.9
					C8T–H2···O4WA ^{v}	0.97	1.92	2.67(3)	132.3

[a] Site occupancy factors (SOFs) are as follows:
10 water molecules are distributed over 16 sites (SOFs in parentheses): O1WA/B (0.2/0.2), O2WA/B/C (0.5/0.3/0.3), O3WA/B (0.3/0.5) and O4WA/B/C (0.5/0.3/0.3) are disordered with TRM outside the α-CD cavity.
O5WA/B/C (0.3/0.3/0.3) are in the cavity formed by the head-to-tail (H2T) stack.
O6WA/B/C (0.2/0.2/0.2) are disordered water sites inside the α-CD cavity.
Twofold disordered TRM–H^+ and one chloride, each was refined to 0.25.

[b] Equivalent positions: (i) –*x*, –*y* + 1, *z*; (ii) –*x* + 1, –*y* + 1, *z*; (iii) –*x* + 0.5, *y* + 0.5, –*z* + 1; (iv) *x*, *y*, *z* – 1; (v) *x*, *y*, *z* + 1; (vi) *x* – 0.5, –*y* + 0.5, –*z* + 1; (vii) –*x* + 1, –*y* + 1, *z* + 1; (viii) –*x* + 0.5, *y* – 0.5, –*z* + 1.

[c] Twofold disordered O61–H group of α-CD (**3**) with occupancy factors of 0.81 and 0.19 for sites A and B, respectively.
Twofold disordered O62–H group of α-CD (**3**) with occupancy factors of 0.87 and 0.13 for sites A and B, respectively.
Twofold disordered O63–H group of α-CD (**3**) with occupancy factors of 0.84 and 0.16 for sites A and B, respectively.

[d] The aromatic plane of TRM–H^+: *Cg*1Z = C2Z–C3Z–C4Z–C4Z^{ii}–C3Z^{ii}–C2Z^{ii}.

(**b**) Intermolecular distances involving water sites and chlorides (Å). [a]

D–H···A	D–H	H···A	D···A	∠(DHA)	D–H···A	D–H	H···A	D···A	∠(DHA)
O1WA···N1Z			2.57(5)		O3WB···O4WA [x]			2.693(15)	
O1WA···Cl1 [i b]			3.32(5)		O3WB···O4WB [x]			2.864(14)	
O1WA···O2WA			2.80(5)		O3WB···O4WC [xi]			2.69(3)	
O1WA···O3WB [ii]			2.96(4)		O4WA···O51 [vii]			2.904(13)	
O1WA···O4WA [iii]			3.20(5)		O4WA···O61A [vii]			3.086(14)	
O1WB···O61B [iv c]			3.21(5)		O4WA···O61B [vii]			3.24(3)	
O1WB···N1Z			2.63(6)		O4WB···O51 [vii]			2.883(11)	
O1WB···N1Z [iv]			2.57(6)		O4WC···O31 [i]			3.12(3)	
O1WB···O2WB			2.80(7)		O4WC···O61A			3.16(3)	
O1WB···O3WA [ii]			3.11(8)		O4WC···O61B			2.75(3)	
O2WA···O1Z			2.92(3)		O4WC···O1Z [i]			2.86(4)	
O2WB···O1Z			2.76(3)		O4WC···N1Z			2.74(4)	
O2WB···O3WB [v]			2.97(3)		O5WA···O62A			2.72(4)	
O2WC···N1Z [vi]			2.90(5)		O5WA···O6WC			2.86(9)	
O2WC···O4WC [vii]			3.15(5)		O5WB···O61A			2.89(5)	
O3WA···O63B [viii]			3.17(4)		O5WB···O62A			2.81(5)	
O3WA···Cl1 [ix]			2.60(3)		O5WB...O6WA			2.63(6)	
O3WA···O4WA [x]			2.72(4)		O5WB···O6WC			3.11(11)	
O3WA···O4WB [x]			3.03(4)		O5WC···O61A [xii]			2.86(5)	
O3WB···O53 [viii]			2.921(9)		O5WC···O63A			2.67(4)	
O3WB···O63A [viii]			2.958(12)		O5WC···O6WA			3.09(6)	
O3WB···O63B [viii]			2.77(3)		O5WC···O6WC			3.00(10)	
O3WB···Cl1 [ix]			2.851(12)						

[a] Site occupancy factors (SOFs) are as follows:
10 water molecules are distributed over 16 sites (SOFs in parentheses): O1WA/B (0.2/0.2), O2WA/B/C (0.5/0.3/0.3), O3WA/B (0.3/0.5) and O4WA/B/C (0.5/0.3/0.3) are disordered with TRM outside the α-CD cavity.
O5WA/B/C (0.3/0.3/0.3) are in the cavity formed by the head-to-tail (H2T) stack.
O6WA/B/C (0.2/0.2/0.2) are disordered water sites inside the α-CD cavity.
Twofold disordered TRM–H^{+} and one chloride, each was refined to 0.25.

[b] Equivalent positions: (i) *x*, *y*, 1 + *z*; (ii) 0.5 – *x*, –0.5 + *y*, 1 – *z*; (iii) 1 – *x*,1 – *y*, 1 + *z*; (iv) 1 – *x*, 1 – *y*, *z*; (v) 0.5 + *x*, 1.5 – *y*, 1 – *z*; (vi) 1 – *x*, 1 – *y*, –1 + *z*; (vii) *x*, *y*, –1 + *z*; (viii) –*x*, 1 – *y*, –1 + *z*; (ix) 0.5 – *x*, 0.5 + *y*, –*z*; (x) –0.5 + *x*, 1.5 – *y*, –*z*; (xi) –0.5 + *x*, 1.5 – *y*, 1 – *z*; (xii) –*x*, 1 – *y*, *z*.

[c] Twofold disordered O61–H group of α-CD (**3**) with occupancy factors of 0.81 and 0.19 for sites A and B, respectively.
Twofold disordered O62–H group of α-CD (**3**) with occupancy factors of 0.87 and 0.13 for sites A and B, respectively.
Twofold disordered O63–H group of α-CD (**3**) with occupancy factors of 0.84 and 0.16 for sites A and B, respectively.

II. Computational data

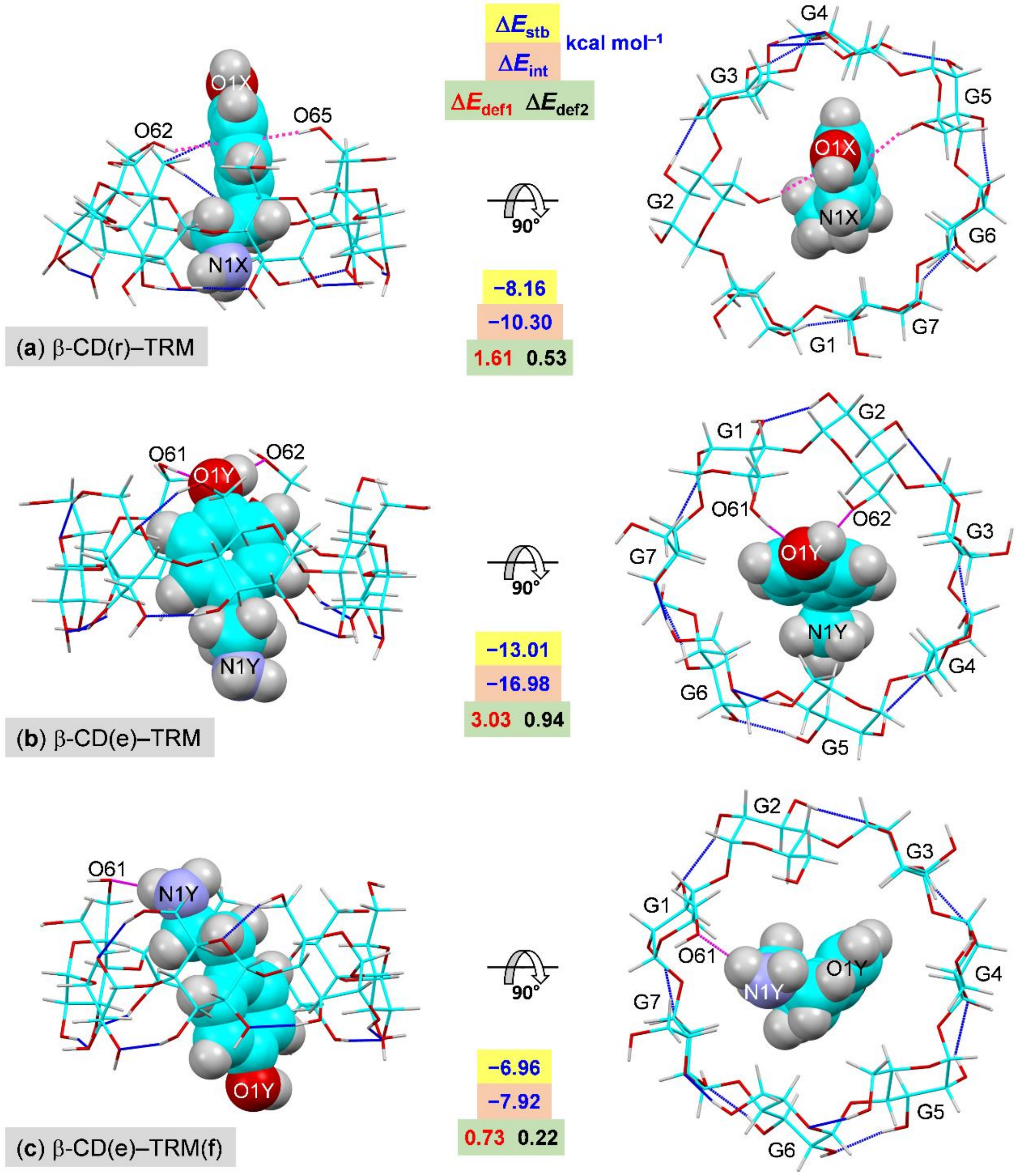

Figure S1. DFT-optimized structures of inclusion complexes (**a**) β-CD(r)−TRM, (**b**) β-CD(e)−TRM, (**c**) β-CD(e)−TRM(f), (**e**) α-CD−TRM(incl), and exclusion complex (**d**) α-CD−TRM(excl); side view (left) and top view (right); see also Figure 5. For comparison, the stabilization, interaction, and deformation energies (ΔE_{stb}, ΔE_{int}, ΔE_{def1}, and ΔE_{def2}) are given in the middle insets; see also Tables S5–S7. The intramolecular O–H···O H-bonds within CDs and intermolecular N/O–H···O/N H-bonds and O–H···π are indicated by blue and magenta connecting lines, respectively. The intramolecular, interglucose C6–H···O5 H-bonds and host–guest C–H···O/N interactions are not shown (Tables S5 and S6).

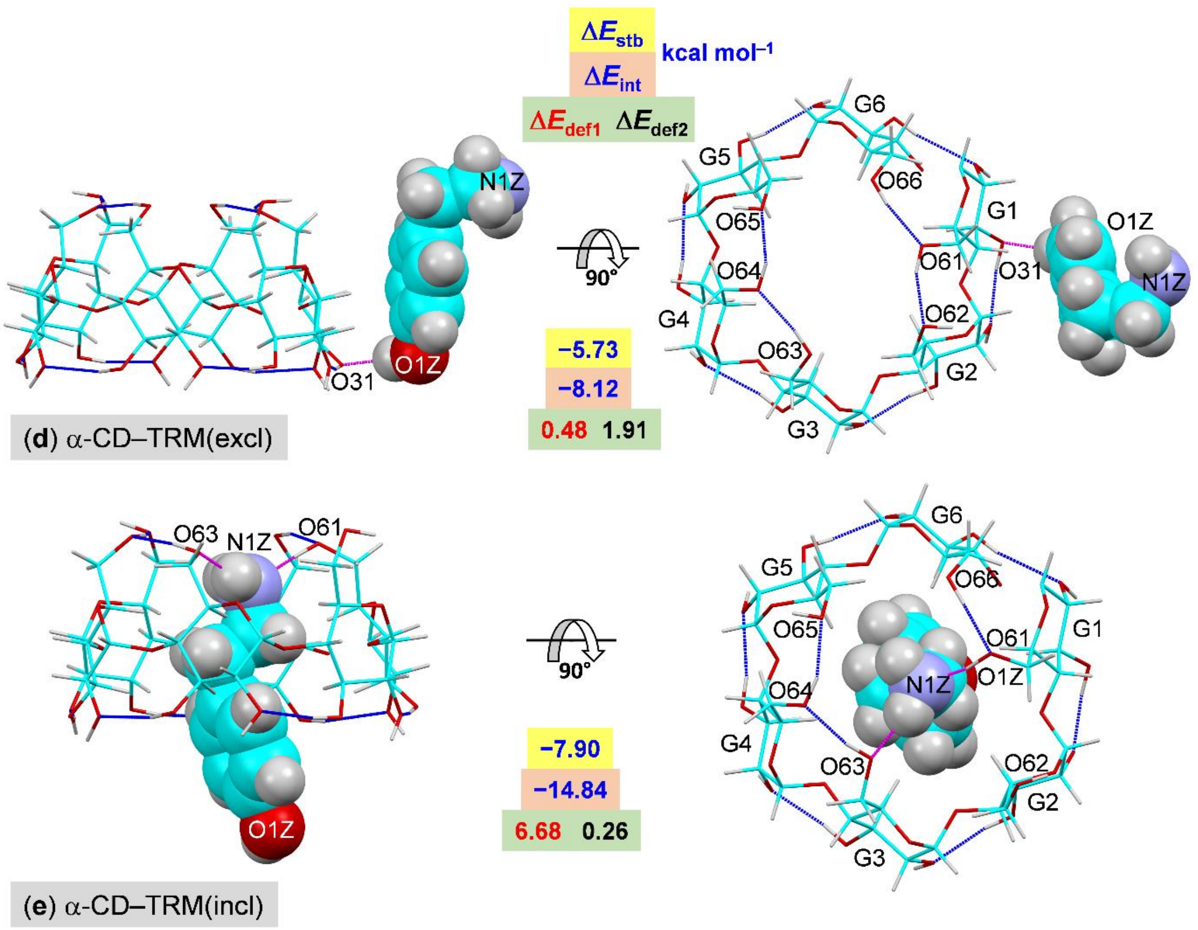

Figure S1. Continued.

Table S5. (**a**) Hydrogen bond parameters of β-CD−TRM base inclusion complexes from different X-ray-derived starting atomic coordinates using DFT full-geometry optimization (Å, º).[a]

D–H···A	D–H	H···A	D···A	∠(DHA)	D–H···A	D–H	H···A	D···A	∠(DHA)
β-CD(r)–TRM[b]			β–CD Distort.	Conform. round					
O21–H···O37	0.98	1.95	2.905	163.1	O62–H···*Cg*[d]	0.97	2.82	3.723	155.9
O32–H···O23	0.98	1.92	2.875	164.4	O65–H···*Cg*	0.97	3.01	3.972	170.1
O33–H···O24	0.98	2.03	2.987	165.1	C61–H1···O52	1.10	2.45	3.475	154.7
O63–H···O54	0.98	1.93	2.894	168.1	C63–H2···O54	1.09	2.64	3.537	139.2
O34–H···O25	0.98	1.93	2.894	165.8	C64–H1···O55	1.09	2.52	3.450	142.2
O35–H···O26	0.98	1.84	2.807	168.5	C67–H2···O51	1.09	2.36	3.300	143.9
O27–H···O36	0.98	2.00	2.857	144.3					
β-CD(e)–TRM[b]				Elliptical	β-CD(e)–TRM(f)[b]			Distorted	round
O21–H···O37	0.98	2.34	3.231	151.2	O21–H···O37	0.98	2.04	2.933	150.4
O22–H···O31	0.98	1.99	2.929	158.8	O22–H···O31	0.98	2.05	2.974	157.7
O32–H···O23	0.98	1.97	2.91	161.9	O32–H···O23	0.98	1.95	2.906	164.2
O33–H···O24	0.98	1.98	2.946	167.6	O33–H···O24	0.98	1.93	2.903	169.6
O25–H···O34	0.98	2.23	3.078	145.1	O25–H···O34	0.98	2.21	3.025	140.7
O35–H···O26	0.98	1.98	2.939	167.8	O35–H···O26	0.98	1.97	2.929	165.7
O65–H···O56	0.98	2.02	2.951	159.6	O65–H···O56	0.98	2.04	2.968	157.7
O36–H···O27	0.98	1.96	2.917	164.8	O36–H···O27	0.98	1.96	2.919	164.3
O66–H···O57	0.97	2.07	3.013	162.1	O66–H···O57	0.97	2.07	3.009	162.5
O61–H···O1Y	0.98	1.94	2.908	172.2	N1Y–H1···O61	1.02	2.28	3.296	172.7
O1Y–H···O62	0.99	1.83	2.788	162.0	C61–H2···O52	1.09	2.21	3.157	144.7
C52–H···*Cg*[c]	1.10	3.48	4.510	157.7	C63–H2···O64	1.09	2.62	3.421	129.1
C55–H···*Cg*	1.10	3.77	4.723	146.4	C64–H1···O55	1.09	2.60	3.573	148.3
C61–H1···O52	1.09	2.47	3.422	144.8	C66–H2···O57	1.09	2.64	3.565	142.0
C64–H1···O55	1.09	2.52	3.450	142.1	C67–H2···O51	1.09	2.55	3.495	144.2
C67–H2···O51	1.09	2.58	3.506	141.8	C37–H···N1Y	1.10	2.66	3.683	154.4
					C6Y–H···O64	1.09	2.45	3.501	162.3

[a] DFT energy minimization in vacuum at the B3LYP/6-31+G(d)/4-31G level, see also Figure 6 and Table S7.
[b] X-ray-derived structures were used as starting models with two plausible inclusion modes:
β-CD(r)–TRM—the round β-CD
β-CD(e)–TRM—the elliptical β-CD
β-CD(e)–TRM(f)—the elliptical β-CD embedding the 180º-flipped TRM
[c] The aromatic plane of TRM: *Cg*1X = C1X–C2X–C3X–C4X–C5X–C6X.
[d] The aromatic plane of TRM: *Cg*1Y = C1Y–C2Y–C3Y–C4Y–C5Y–C6Y.

(**b**) Selected structural parameters of β-CD−TRM base inclusion complex from different X-ray-derived starting atomic coordinates using DFT full-geometry optimization, in comparison to those of the original X-ray structures.

G	Tilt angle (º)[a]					O4 deviation (Å)[b]				
n	(**1**)	(**2**)	β-CD(r) –TRM	β-CD(e) –TRM	β-CD(e) –TRM(f)	(**1**)	(**2**)	β-CD(r) –TRM	β-CD(e) –TRM	β-CD(e) –TRM(f)
1	10.9(3)	10.0(2)	8.8	18.9	12.0	0.305(3)	–0.339(2)	0.017	–0.367	–0.229
2	19.0(3)	23.5(1)	34.4	30.6	26.1	–0.168(3)	0.088(2)	0.268	–0.089	–0.044
3	4.3(3)	8.1(1)	11.5	6.4	3.9	–0.186(3)	0.367(2)	–0.294	0.363	0.248
4	17.7(3)	13.1(2)	4.1	6.0	5.0	0.229(3)	–0.290(2)	0.002	–0.067	–0.072
5	25.3(3)	35.4(2)	16.0	30.4	25.5	0.066(3)	–0.216(2)	0.204	–0.291	–0.208
6	8.6(3)	13.8(1)	6.4	20.5	17.4	–0.217(2)	0.380(2)	–0.075	0.122	0.153
7	9.1(1)	11.2(1)	4.5	2.5	2.3	–0.029(3)	0.011(2)	–0.123	0.327	0.152

[a] Interplanar angle of the plane through C1(*n*), C4(*n*), O4(*n*) and O4(*n* – 1) against the O4 plane.
[b] Deviation of glycosidic O4 atoms from the least-squares plane through the seven O4 atoms.

Table S6. (**a**) Hydrogen bond parameters of α-CD−TRM base exclusion and inclusion complexes derived from DFT full-geometry optimization (Å, º). [a]

D–H···A	D–H	H···A	D···A	∠(DHA)	D–H···A	D–H	H···A	D···A	∠(DHA)
α-CD–TRM(excl) [b]			α–CD	Conform. Round	α-CD–TRM(incl) [b]				Round
O31–H···O22	0.98	2.00	2.962	165.7	O31–H···O22	0.98	1.91	2.873	167.5
O61–H···O62	0.98	1.95	2.887	159.5	O32–H···O23	0.98	2.05	3.018	167.4
O32–H···O23	0.98	1.99	2.953	166.7	O33–H···O24	0.98	2.04	3.004	166.6
O33–H···O24	0.98	2.25	3.206	165.8	O63–H···O64	0.98	1.86	2.819	165.1
O63–H···O64	0.98	1.98	2.947	169.0	O34–H···O25	0.98	2.13	3.089	165.3
O34–H···O25	0.98	2.09	3.050	167.0	O64–H···O65	0.98	1.97	2.908	159.9
O64–H···O65	0.98	1.96	2.891	158.5	O35–H···O26	0.98	1.92	2.891	169.5
O35–H···O26	0.98	1.99	2.955	166.5	O36–H···O21	0.98	2.31	3.263	164.9
O36–H···O21	0.98	2.36	3.310	164.7	O66–H···O61	0.98	1.89	2.851	166.1
O66–H···O61	0.98	1.97	2.938	167.9	N1Z–H2···O63	1.03	2.11	3.109	165.3
O1Z–H···O31	0.98	1.95	2.877	156.7	O61–H···N1Z	1.00	1.86	2.846	167.1
C41–H···*Cg*1Z [c]	1.09	3.145	4.198	161.5	C62–H1···O53	1.09	2.63	3.538	139.8
C12–H···*Cg*1Z [c]	1.09	4.000	2.913	173.5	C63–H1···O54	1.09	2.64	3.464	131.5
C61–H1···O52	1.09	2.56	3.491	142.8	C64–H1···O55	1.09	2.45	3.416	146.0
C63–H1···O54	1.09	2.29	3.302	152.4	C66–H1···O51	1.09	2.33	3.307	148.6
C64–H1···O55	1.09	2.48	3.434	145.5					
C65–H1···O66	1.09	2.61	3.667	164.0					
C66–H1···O51	1.09	2.25	3.255	152.5					

[a] DFT energy minimization in vacuum at the B3LYP/6-31+G(d)/4-31G level, see also Figure 6 and Table S7.
[b] X-ray-derived structures were used as starting models with two plausible complexation modes:
α-CD–TRM(excl)—the exclusion complex
α-CD–TRM(incl)—the inclusion complex
[c] The aromatic plane of TRM: *Cg*1Z = C1Z–C2Z–C3Z–C4Z–C5Z–C6Z.

(**b**) Selected structural parameters of α-CD−TRM base exclusion and inclusion complexes derived from DFT full-geometry optimization, in comparison to those of the original X-ray structure.

G	Tilt angle (º) [a]			O4 deviation (Å) [b]		
n	(**3**)	α-CD–TRM(excl)	α-CD–TRM(incl)	(**3**)	α-CD–TRM(excl)	α-CD–TRM(incl)
1	11.2(1)	17.3	17.8	–0.016(2)	0.088	0.117
2	9.5(1)	15.3	5.9	–0.001(2)	0.058	–0.023
3	10.7(1)	14.6	14.6	0.018(2)	–0.137	–0.044
4	11.2(1)	15.1	11.0	–0.016(2)	0.067	0.010
5	9.5(1)	17.8	16.2	–0.001(2)	0.079	0.086
6	10.7(1)	15.3	9.6	0.018(2)	–0.156	–0.146

[a] Interplanar angle of the plane through C1(n), C4(n), O4(n) and O4(n – 1) against the O4 plane.
[b] Deviation of glycosidic O4 atoms from the least-squares plane through the six O4 atoms.

Table S7. Stabilization and interaction energies of β-CD and α-CD complex with TRM base from different X-ray-derived starting atomic coordinates using DFT full-geometry optimization and single-point energy calculation. [a]

Complex	β-CD(r)–TRM	β-CD(r)–TRM	β-CD(e)–TRM	β-CD(e)–TRM	β-CD(e)–TRM(f)	α-CD–TRM(excl)	α-CD–TRM(incl)
	Single-point	Full-opt	Single-point	Full-opt	Full-opt	Full-opt	Full-opt
E_{cpx} [b]	−4713.72696	−4714.01085	−4713.87967	−4714.02246	−4714.01237	−4103.61398	−4103.61689
E_{CD_opt} [c]		−4272.95713		−4272.96108	−4272.96061	−3662.56306	−3662.56296
E_{TRM_opt}		−441.04072		−441.04065	−441.04066	−441.04179	−441.04135
E_{CD_sp}	−4272.72092	−4272.95456	−4272.84633	−4272.95625	−4272.95944	−3662.56230	−3662.55231
E_{TRM_sp}	−441.00746	−441.03987	−441.02606	−441.03916	−441.04031	−441.03875	−441.04093
ΔE_{stb} (Hartree) [d]		−0.01301		−0.02073	−0.01110	−0.00913	−0.01258
ΔE_{stb} (kcal mol^{-1})		−8.16		−13.01	−6.96	−5.73	−7.90
$\Delta\Delta E_{stb}$ (kcal mol^{-1}) [f]		4.85		0	6.05	2.17	0
ΔE_{int} (Hartree) [e]	0.00142	−0.01642	−0.00728	−0.02705	−0.01262	−0.01293	−0.02364
ΔE_{int} (kcal mol^{-1})	0.89	−10.30	−4.57	−16.98	−7.92	−8.12	−14.84
$\Delta\Delta E_{int}$ (kcal mol^{-1}) [g]		6.68		0	9.06	6.72	0
ΔE_{def1} (Hartree) [h]		0.00256		0.00482	0.00117	0.00076	0.01065
ΔE_{def1} (kcal mol^{-1})		1.61		3.03	0.73	0.48	6.68
ΔE_{def2} (Hartree) [i]		0.00085		0.00149	0.00035	0.00304	0.00041
ΔE_{def2} (kcal mol^{-1})		0.53		0.94	0.22	1.91	0.26
No. and type of host–guest interactions	1 CH···π	2 O–H···π	2 C–H···π	2 O–H···O<br>2 C–H···π	1 N–H···O<br>1 C–H···N<br>1 C–H···O	1 O–H···O<br>2 C–H···π	1 N–H···O<br>1 O–H···N

[a] DFT/B3LYP calculations in the gas phase with mixed basis sets 4-31G for C atoms and 6-31+G(d) for H, O atoms were carried out using program GAUSSIAN09 [4]. X-ray-derived structures were used as starting models, see also Figure 6 and Tables S5, S6.

β-CD(r)–TRM—the round β-CD

β-CD(e)–TRM—the elliptical β-CD

β-CD(e)–TRM(f)—the elliptical β-CD embedding the 180°-flipped TRM

α-CD–TRM(excl)—the exclusion complex

α-CD–TRM(incl)— the inclusion complex

[b] Original unit of E is Hartree (1 H = 627.5 kcal mol^{-1}).

[c] E_{CD_opt} in vacuum of the uncomplexed β-CD·12H_2O [2] and α-CD·6H_2O [3] are −4272.96662 and −3662.52433 H (excluded water of hydration), respectively.

[d,e] Stabilization energy, $\Delta E_{stb} = E_{cpx} - (E_{CD\_opt} + E_{TRM\_opt})$
Interaction energy, $\Delta E_{int} = E_{cpx} - (E_{CD\_sp} + E_{TRM\_sp})$,
where E_{cpx}, E_{CD_opt} and E_{TRM_opt} are the respective molecular energies from full optimization of complex, host CD and guest TRM; E_{CD_sp} and E_{TRM_sp} are the corresponding single-point energies in the complexed states.

[f,g] Relative stabilization energy and relative interaction energy ($\Delta\Delta E_{stb}$ and $\Delta\Delta E_{int}$) calculated using the most stable complex as a reference.

[h,i] Deformation energies of CD (ΔE_{def1}) and TRM (ΔE_{def2}) are expressed as:
$\Delta E_{def1} = E_{CD\_sp} - E_{CD\_opt}$, $\Delta E_{def2} = E_{TRM\_sp} - E_{TRM\_opt}$, and $\Delta E_{stb} = \Delta E_{int} + \Delta E_{def1} + \Delta E_{def2}$.

Table S8. Dispersion- and BSSE-corrected interaction energies of β-CD and α-CD complex with TRM base from DFT/B97D calculations. [a]

Complex	β-CD(r)–TRM	β-CD(e)–TRM	β-CD(e)–TRM(f)	α-CD–TRM(excl)	α-CD–TRM(incl)
B97D					
E_{cpx}	−4714.08750	−4714.10139	−4714.08957	−4103.67862	−4103.70547
E_{CD_sp}	−4272.87861	−4272.88467	−4272.88648	−3662.49349	−3662.48247
E_{TRM_sp}	−441.16433	−441.16302	−441.16455	−441.16296	−441.16494
ΔE_{int} (Hartree)	−0.04457	−0.05369	−0.03854	−0.02217	−0.05807
ΔE_{int} (kcal mol^{-1})	−27.97	−33.69	−24.18	−13.91	−36.44
$\Delta\Delta E_{int}$ (kcal mol^{-1}) [d]	5.72	0	9.51	22.53	0
B97D+BSSE					
E_{cpx_BSSE}	−4714.08119	−4714.09419	−4714.08368	−4103.67598	−4103.69837
$E_{CD(cpx)\_sp}$	−4272.88227	−4272.88866	−4272.88979	−3662.49464	−3662.48641
$E_{TRM(cpx)\_sp}$	−441.16698	−441.16623	−441.16714	−441.16445	−441.16810
ΔE_{BSSE} (Hartree) [b]	0.00631	0.00720	0.00589	0.00264	0.00710
ΔE_{BSSE} (kcal mol^{-1})	3.96	4.52	3.70	1.66	4.46
% of ΔE_{BSSE} in ΔE_{int}	14.2	13.4	15.3	11.9	12.2
ΔE_{int_BSSE} (Hartree) [c]	−0.03825	−0.04650	−0.03265	−0.01953	−0.05097
ΔE_{int_BSSE} (kcal mol^{-1})	−24.00	−29.18	−20.49	−12.26	−31.98
$\Delta\Delta E_{int\_BSSE}$ (kcal mol^{-1}) [d]	5.18	0	8.69	19.72	0

[a] DFT/B97D calculations in the gas phase with 6-31+G(d,p) for all atoms were carried out using program GAUSSIAN09 [4]. The DFT/B3LYP-optimized structures were used for single-point energy calculations, see also Table S7.

[b] The basis set superposition error (BSSE) energy by counterpoise correction [5],

$$\Delta E_{BSSE} = E_{cpx\_BSSE} - E_{cpx} = [E_{CD\_sp} - E_{CD(cpx)\_sp}] + [E_{TRM\_sp} - E_{TRM(cpx)\_sp}]$$

where E_{cpx_BSSE} and E_{cpx} are corrected and uncorrected complex energies, respectively.

[c] BSSE-corrected interaction energy, $\Delta E_{int\_BSSE} = E_{cpx} - (E_{CD(cpx)\_sp} + E_{TRM(cpx)\_sp}) = \Delta E_{int} + \Delta E_{BSSE}$

where $E_{CD(cpx)\_sp}$ and $E_{TRM(cpx)\_sp}$ are energies of two components in the complex.

[d] Relative interaction energies ($\Delta\Delta E_{int}$ and $\Delta\Delta E_{int\_BSSE}$)

are calculated using the most stable complexes of β-CD(e)–TRM and α-CD–TRM(incl) as references.

III. References

1. Cremer, D.; Pople, J.A. General Definition of Ring Puckering Coordinates. *J. Am. Chem. Soc.* **1975**, *97*, 1354–1358, doi:10.1021/ja00839a011.
2. Lindner, K.; Saenger, W. Crystal and Molecular Structure of Cyclohepta-Amylose Dodecahydrate. *Carbohydr. Res.* **1982**, *99*, 103–115, doi:10.1016/S0008-6215(00)81901-1.
3. Manor, P.C.; Saenger, W. Topography of Cyclodextrin Inclusion Complexes. III. Crystal and Molecular Structure of Cyclohexaamylose Hexahydrate, the Water Dimer Inclusion Complex. *J. Am. Chem. Soc.* **1974**, *96*, 3630–3639, doi:10.1021/ja00818a042.
4. Frisch, M.J.; Trucks, G.W.; Schlegel, H.B.; Scuseria, G.E.; Robb, M.A.; Cheeseman, J.R.; ...; Nakatsuji, H. GAUSSIAN09 2009.
5. Boys, S.F.; Bernardi, F. The Calculation of Small Molecular Interactions by the Differences of Separate Total Energies. Some Procedures with Reduced Errors. *Mol. Phys.* **1970**, *19*, 553–566, doi:10.1080/00268977000101561.
